# Supplementary material for: Simultaneous carbon catabolite repression governs sugar and aromatic co-utilization in Pseudomonas putida M2
Source: Appl Environ Microbiol. 2023 Sep 19;89(10):e00852-23. doi: 10.1128/aem.00852-23 (PMC10617552; doi:10.1128/aem.00852-23)
Supplement: Supplemental material — Figures and tables. [file aem.00852-23-s0001.docx]

# **Supporting Information**

# **Simultaneous carbon catabolite repression governs sugar and aromatic co-utilization in *Pseudomonas putida* M2**

## Shilva Shrestha,^a,b^ Deepika Awasthi,^a,b^ Yan Chen,^a,b^ Jennifer Gin,^a,b^ Christopher J. Petzold,^a,b^ Paul D. Adams,^a,c^ ,Blake A. Simmons,^a,b^ Steven W. Singer ^a,b^*

^a^Joint BioEnergy Institute, Emeryville, California, USA

^b^Biological Systems and Engineering Division, Lawrence Berkeley National Laboratory, Berkeley, California, USA

^c^Molecular Biophysics and Integrated Bioimaging Division, Lawrence Berkeley National Laboratory, Berkeley, California, USA

*^*^* Address correspondence to: Steven W. Singer, [swsinger@lbl.gov](mailto:swsinger@lbl.gov)

## **TABLES**

| **Table S1.** Growth characteristics of wild type *Pseudomonas putida* M2 in single carbon source. | | | | |
| --- | --- | --- | --- | --- |
| Substrate | Growth rate (h^-1^) | Doubling time (h) | Substrate consumption  (g L^-1^ h^-1^) |  |
|  |  |  |  |  |
| Glucose | 0.24 ± 0.01 | 2.90 ± 0.10 | 0.88 ± 0.08 |  |
| Xylose | 0.33 ± 0.00 | 2.10 ± 0.02 | 0.60 ± 0.01 |  |
| *p*-coumarate | 0.33 ± 0.00 | 2.10 ± 0.02 | 0.38 ± 0.10 |  |
| 4-hydroxybenzoate | 0.28 ± 0.02 | 2.46 ± 0.20 | 0.25 ± 0.07 |  |
| Ferulate | 0.15 ± 0.01 | 4.69 ± 0.24 | 0.21 ± 0.10 |  |
| Vanillate | 0.27 ± 0.00 | 2.54 ± 0.03 | 0.15 ± 0.01 |  |

| **Table S2.** Growth rate (h^-1^) of wild-type *P. putida* M2 and CRISPRi strains including control in a mixture of sugar + aromatic compound. | | | | | | | | | | |
| --- | --- | --- | --- | --- | --- | --- | --- | --- | --- | --- |
| Substrate/  Strain | Glucose + | | | | | Xylose + | | | | |
|  | *p*-coumarate | 4-hydroxybenzoate | Ferulate | Vanillate | *p*-coumarate | | 4-hydroxybenzoate | Ferulate | Vanillate | |
| wild type | 0.22 ± 0.01 | 0.25 ± 0.01 | 0.20 ± 0.02 | 0.14 ± 0.03 | 0.24 ± 0.02 | | 0.25 ± 0.01 | 0.26 ± 0.01 | 0.22 ± 0.01 | |
| control | 0.11 ± 0.02 | N.D | 0.16 ± 0.06 | N.D | 0.14 ± 0.01 | | N.D | N.D | N.D | |
| crc-1 | 0.19 ± 0.01 | N.D | 0.20 ± 0.01 | N.D | 0.12 ± 0.00 | | N.D | N.D | N.D | |
| crc-2 | 0.19 ± 0.03 | N.D | 0.23 ± 0.03 | N.D | 0.13 ± 0.00 | | N.D | N.D | N.D | |
| crc-3 | 0.17 ± 0.03 | N.D | 0.22 ± 0.08 | N.D | 0.12 ± 0.00 | | N.D | N.D | N.D | |
| Note N.D. refers to not determined. | | | | | | | | | |  |

| **Table S3.** Doubling time (h) of wild-type *P. putida* M2 and CRISPRi strains including control in a mixture of sugar + aromatic compound. | | | | | | | | |  |  |
| --- | --- | --- | --- | --- | --- | --- | --- | --- | --- | --- |
| Substrate/  Strain | Glucose + | | | | Xylose + | | | | |  |
|  | *p*-coumarate | 4-hydroxybenzoate | Ferulate | Vanillate | *p*-coumarate | 4-hydroxybenzoate | Ferulate | Vanillate |  |  |
| wild type | 3.11 ± 0.18 | 2.76 ± 0.09 | 3.42 ± 0.38 | 4.91 ± 0.95 | 2.92 ± 0.24 | 2.76 ± 0.09 | 2.70 ± 0.01 | 3.12 ± 0.11 |  |  |
| control | 6.16 ± 1.05 | N.D | 4.77 ± 1.88 | N.D | 5.10 ± 0.25 | N.D | N.D | N.D |  |  |
| crc-1 | 3.67 ± 0.30 | N.D | 3.55 ± 0.11 | N.D | 5.58 ± 0.06 | N.D | N.D | N.D |  |  |
| crc-2 | 3.72 ± 0.54 | N.D | 3.06 ± 0.48 | N.D | 5.54 ± 0.11 | N.D | N.D | N.D |  |  |
| crc-3 | 4.23 ± 0.86 | N.D | 3.49 ± 1.22 | N.D | 5.66 ± 0.18 | N.D | N.D | N.D |  |  |
| Note N.D. refers to not determined. | | | | | | | | | | |

| **Table S4.** Substrate consumption rate (g L^-1^ h^-1^) of sugar of wild-type *P. putida* M2 and CRISPRi strains including control in a mixture of sugar + aromatic compound. | | | | | | | | |  |  |
| --- | --- | --- | --- | --- | --- | --- | --- | --- | --- | --- |
| Substrate/  Strain | Glucose + | | | | Xylose + | | | | |  |
|  | *p*-coumarate | 4-hydroxybenzoate | Ferulate | Vanillate | *p*-coumarate | 4-hydroxybenzoate | Ferulate | Vanillate | |  |
| wild type | 0.63 ± 0.06 | 0.29 ± 0.02 | 0.61 ± 0.40 | 0.68 ± 0.11 | 1.07 ± 0.43 | 0.72 ± 0.02 | 1.07 ± 0.43 | 0.72 ± 0.05 | |  |
| control | 0.55 ± 0.10 | N.D | 0.29 ± 0.07 | N.D | 0.58 ± 0.41 | N.D | N.D | N.D | |  |
| crc-1 | 1.32 ± 0.18 | N.D | 0.33 ± 0.06 | N.D | 0.66 ± 0.06 | N.D | N.D | N.D | |  |
| crc-2 | 0.73 ± 0.09 | N.D | 0.28 ± 0.05 | N.D | 0.43 ± 0.25 | N.D | N.D | N.D | |  |
| crc-3 | 1.96 ± 0.29 | N.D | 0.26 ± 0.07 | N.D | 1.01 ± 0.21 | N.D | N.D | N.D | |  |
| Note N.D. refers to not determined. | | | | | | | | | | |

| \| **Table S5.** Substrate consumption rate (g L^-1^ h^-1^) of aromatic compound of wild-type *P. putida* M2 and CRISPRi strains including control in a mixture of sugar + aromatic compound. \| \| \| \| \| \| \| \| \| \| \| --- \| --- \| --- \| --- \| --- \| --- \| --- \| --- \| --- \| --- \| \| Substrate/  Strain \| Glucose + \| \| \| \| Xylose + \| \| \| \| \| \| *p*-coumarate \| 4-hydroxybenzoate \| Ferulate \| Vanillate \| *p*-coumarate \| 4-hydroxybenzoate \| Ferulate \| Vanillate \| \| wild type \| 0.14 ± 0.01 \| 0.10 ± 0.00 \| 0.09 ± 0.01 \| 0.16 ± 0.11 \| 0.28 ± 0.04 \| 0.17 ± 0.02 \| 0.28 ± 0.04 \| 0.07 ± 0.01 \| \| control \| 0.08 ± 0.01 \| N.D \| 0.06 ± 0.05 \| N.D \| 0.19 ± 0.01 \| N.D \| N.D \| N.D \| \| crc-1 \| 0.17 ± 0.01 \| N.D \| 0.06 ± 0.01 \| N.D \| 0.19 ± 0.01 \| N.D \| N.D \| N.D \| \| crc-2 \| 0.14 ± 0.03 \| N.D \| 0.08 ± 0.03 \| N.D \| 0.14 ± 0.00 \| N.D \| N.D \| N.D \| \| crc-3 \| 0.12 ± 0.06 \| N.D \| 0.09 ± 0.01 \| N.D \| 0.14 ± 0.01 \| N.D \| N.D \| N.D \| \| Note N.D. refers to not determined. \| \| \| \| \| \| \| \| \| \| |
| --- | --- | --- | --- | --- | --- | --- | --- | --- | --- | --- | --- | --- | --- | --- | --- | --- | --- | --- | --- | --- | --- | --- | --- | --- | --- | --- | --- | --- | --- | --- | --- | --- | --- | --- | --- | --- | --- | --- | --- | --- | --- | --- | --- | --- | --- | --- | --- | --- | --- | --- | --- | --- | --- | --- | --- | --- | --- | --- | --- | --- | --- | --- | --- | --- | --- | --- | --- | --- | --- | --- | --- | --- | --- | --- | --- | --- | --- | --- | --- | --- | --- | --- | --- |

| **Table S6.** Bacterial strains grown under different carbon sources submitted for shotgun proteomics. | |
| --- | --- |
| Strain | Carbon source |
| M2 | glucose, xylose, p-coumarate, 4-hydroxybenzoate, ferulate, vanillate, glucose + *p*-coumarate, glucose + 4-hydroxybenzoate, glucose + ferulate, glucose + vanillate, xylose + *p*-coumarate, xylose + 4-hydroxybenzoate, xylose + ferulate, or xylose + vanillate |
| KT2440 | glucose, *p*-coumarate, 4-hydroxybenzoate, ferulate, vanillate, glucose +  *p*-coumarate, glucose + 4-hydroxybenzoate, glucose + ferulate, or glucose + vanillate |
| M2 crc-1, crc-2, crc-3, control | glucose + *p*-coumarate |

| **Table S7.** Significantly (p ≤ 0.05) lower abundance proteins in *P. putida* M2 proteome in media containing glucose + *p*-coumarate with corresponding log_2_ fold change when compared with that in *p*-coumarate only. Only proteins with log_2_ FC ≥ 0.5 value are shown. | | | |
| --- | --- | --- | --- |
| **Protein name** | **Locus_tag** | **Log_2_ FC** | **p-value** |
| Isocitrate lyase | Ga0436255_01_4401307_4402632 | -3.5 | 3.5E-04 |
| arylsulfatase | Ga0436255_01_258380_260050 | -3.3 | 9.5E-04 |
| 3-carboxy-cis,cis-muconate cycloisomerase | Ga0436255_01_1321597_1322949 | -3.3 | 2.1E-04 |
| acetyl-CoA synthetase | Ga0436255_01_3940845_3942806 | -3.3 | 2.1E-04 |
| electron-transferring-flavoprotein dehydrogenase | Ga0436255_01_4243592_4245256 | -2.8 | 1.5E-04 |
| alkylation response protein AidB-like acyl-CoA dehydrogenase | Ga0436255_01_261038_262777* | -2.7 | 1.4E-03 |
| feruloyl-CoA synthase | Ga0436255_01_264030_265913 | -2.5 | 7.4E-04 |
| Ga0436255_01_5414412_5415977 | Ga0436255_01_5414412_5415977 | -2.5 | 1.8E-03 |
| 4-carboxymuconolactone decarboxylase | Ga0436255_01_1320385_1320783 | -2.5 | 2.6E-02 |
| hypothetical protein | Ga0436255_01_4777849_4778391 | -2.1 | 2.2E-03 |
| aldehyde dehydrogenase | Ga0436255_01_1761115_1762635 [2] | -2.1 | 1.5E-04 |
| 2,5-diketo-D-gluconate reductase B | Ga0436255_01_52955_53779 | -2.0 | 1.6E-02 |
| p-hydroxybenzoate 3-monooxygenase | Ga0436255_01_348086_349273 | -1.9 | 3.5E-04 |
| capsular exopolysaccharide synthesis family protein | Ga0436255_01_4871871_4872677 | -1.9 | 9.1E-03 |
| Ga0436255_01_5407420_5408097 | Ga0436255_01_5407420_5408097 | -1.9 | 8.9E-03 |
| malate synthase | Ga0436255_01_2838056_2840233 | -1.7 | 1.4E-04 |
| 3-oxoadipate enol-lactonase | Ga0436255_01_1320794_1321585 | -1.7 | 4.9E-04 |
| 5-methyltetrahydropteroyltriglutamate--homocysteine methyltransferase | Ga0436255_01_1535993_1538293 | -1.6 | 1.3E-02 |
| methyl-accepting chemotaxis protein | Ga0436255_01_559450_560928 [24] | -1.5 | 1.2E-02 |
| branched-chain amino acid transport system substrate-binding protein | Ga0436255_01_1169501_1170616 | -1.5 | 1.4E-04 |
| acyl-CoA reductase-like NAD-dependent aldehyde dehydrogenase | Ga0436255_01_265990_267438 | -1.4 | 2.9E-02 |
| branched-chain amino acid transport system substrate-binding protein | Ga0436255_01_3043696_3044832* | -1.4 | 3.2E-02 |
| 3-oxoadipyl-CoA thiolase | Ga0436255_01_1324437_1325639 | -1.3 | 1.2E-05 |
| glutamate/aspartate transport system substrate-binding protein | Ga0436255_01_1227546_1228463 | -1.3 | 9.7E-03 |
| trans-feruloyl-CoA hydratase/vanillin synthase | Ga0436255_01_267514_268344 | -1.3 | 1.5E-02 |
| catalase-peroxidase | Ga0436255_01_49281_51536 | -1.2 | 1.0E-02 |
| nicotinamidase-related amidase | Ga0436255_01_3254337_3254957 | -1.1 | 3.2E-04 |
| hypothetical protein | Ga0436255_01_3301709_3303073 | -1.1 | 8.0E-03 |
| uroporphyrin-3 C-methyltransferase | Ga0436255_01_2145309_2146409 | -1.1 | 1.2E-02 |
| 3-oxoadipate CoA-transferase alpha subunit | Ga0436255_01_5391009_5391704 | -1.1 | 3.5E-03 |
| Ga0436255_01_1671621_1673171 | Ga0436255_01_1671621_1673171 | -1.0 | 4.3E-02 |
| poly(hydroxyalkanoate) granule-associated protein | Ga0436255_01_1915998_1916762 | -1.0 | 1.9E-02 |
| glutamate dehydrogenase | Ga0436255_01_719906_724771 | -1.0 | 5.7E-04 |
| protocatechuate 3,4-dioxygenase beta subunit | Ga0436255_01_1558507_1559226 | -1.0 | 3.3E-02 |
| beta-alanine--pyruvate transaminase | Ga0436255_01_3143828_3145174 | -0.9 | 1.6E-02 |
| Ga0436255_01_4640393_4642417 | Ga0436255_01_4640393_4642417 | -0.8 | 3.6E-03 |
| small subunit ribosomal protein S12 | Ga0436255_01_2953505_2953876 | -0.8 | 1.6E-03 |
| isocitrate dehydrogenase | Ga0436255_01_4374278_4375534 | -0.7 | 4.0E-03 |
| adhesin transport system outer membrane protein | Ga0436255_01_3898785_3900158 | -0.7 | 9.6E-03 |
| succinate dehydrogenase / fumarate reductase flavoprotein subunit | Ga0436255_01_4255131_4256903 | -0.6 | 2.7E-03 |
| DNA-binding protein HU-alpha | Ga0436255_01_2283538_2283837* | -0.6 | 1.6E-02 |

| **Table S8.** Significantly (p ≤ 0.05) lower abundance proteins in *P. putida* M2 proteome in media containing glucose + 4-hydroxybenzoate with corresponding log_2_ fold change when compared with that in 4-hydroxybenzoate only. Only proteins with log_2_ FC ≥ 0.5 value are shown. | | | | |
| --- | --- | --- | --- | --- |
| **Protein name** | **Locus_tag** | | **Log_2_ FC** | **p-value** |
| hypothetical protein | Ga0436255_01_3301709_3303073 | | -2.9 | 3.8E-03 |
| electron-transferring-flavoprotein dehydrogenase | Ga0436255_01_4243592_4245256 | | -2.9 | 1.1E-03 |
| aldehyde dehydrogenase | Ga0436255_01_1761115_1762635 [2] | | -2.8 | 3.5E-03 |
| RHH-type proline utilization regulon transcriptional repressor/proline dehydrogenase/delta 1-pyrroline-5-carboxylate dehydrogenase | Ga0436255_01_1840552_1844505 [2] | | -2.6 | 1.1E-02 |
| 4-carboxymuconolactone decarboxylase | Ga0436255_01_1320385_1320783 | | -2.5 | 2.7E-03 |
| Ga0436255_01_5414412_5415977 | Ga0436255_01_5414412_5415977* | | -2.5 | 4.4E-04 |
| uroporphyrin-3 C-methyltransferase | Ga0436255_01_2145309_2146409 | | -2.4 | 7.0E-03 |
| branched-chain amino acid transport system substrate-binding protein | Ga0436255_01_3043696_3044832* | | -2.4 | 6.1E-04 |
| methyl-accepting chemotaxis protein | Ga0436255_01_559450_560928 [24] | | -2.4 | 5.1E-04 |
| catalase-peroxidase | Ga0436255_01_49281_51536 | | -2.2 | 1.6E-02 |
| 2,4-dienoyl-CoA reductase-like NADH-dependent reductase (Old Yellow Enzyme family) | Ga0436255_01_3619914_3621020 | | -2.2 | 4.8E-05 |
| malate synthase | Ga0436255_01_2838056_2840233 | | -2.1 | 3.4E-03 |
| outer membrane protein OmpA-like peptidoglycan-associated protein | Ga0436255_01_1186969_1187661 | | -2.0 | 4.1E-02 |
| branched-chain amino acid transport system substrate-binding protein | Ga0436255_01_1169501_1170616 | | -2.0 | 4.0E-04 |
| quinoprotein glucose dehydrogenase | Ga0436255_01_1253878_1256289 | | -2.0 | 2.3E-03 |
| general L-amino acid transport system substrate-binding protein | Ga0436255_01_1443964_1444992* | | -1.8 | 1.1E-02 |
| 3-carboxy-cis,cis-muconate cycloisomerase | Ga0436255_01_1321597_1322949 | | -1.8 | 1.7E-03 |
| polar amino acid transport system substrate-binding protein | Ga0436255_01_2744051_2744803* | | -1.8 | 8.6E-04 |
| p-hydroxybenzoate 3-monooxygenase | Ga0436255_01_348086_349273 | | -1.7 | 2.9E-03 |
| carbon starvation protein | Ga0436255_01_1541217_1543283* | | -1.6 | 4.3E-02 |
| Ga0436255_01_4643848_4644516 | Ga0436255_01_4643848_4644516 | | -1.6 | 1.0E-02 |
| gamma-glutamyltranspeptidase/glutathione hydrolase | Ga0436255_01_1562537_1564204* | | -1.6 | 3.4E-03 |
| Ga0436255_01_1671621_1673171 | Ga0436255_01_1671621_1673171* | | -1.5 | 5.8E-03 |
| putative spermidine/putrescine transport system substrate-binding protein | Ga0436255_01_1033189_1034253* | | -1.5 | 7.5E-03 |
| nicotinamidase-related amidase | Ga0436255_01_3254337_3254957* | | -1.4 | 2.5E-03 |
| Ga0436255_01_5407420_5408097 | Ga0436255_01_5407420_5408097* | | -1.4 | 2.3E-02 |
| 2,5-diketo-D-gluconate reductase B | Ga0436255_01_52955_53779 | | -1.4 | 1.0E-02 |
| 3-oxoadipate enol-lactonase | Ga0436255_01_1320794_1321585 | | -1.3 | 4.8E-04 |
| glutamate dehydrogenase | Ga0436255_01_719906_724771 | | -1.2 | 4.1E-04 |
| protocatechuate 3,4-dioxygenase beta subunit | Ga0436255_01_1558507_1559226 | | -1.2 | 1.6E-02 |
| TRAP transporter TAXI family solute receptor | Ga0436255_01_761820_762770* | | -1.2 | 6.1E-03 |
| poly(hydroxyalkanoate) granule-associated protein | Ga0436255_01_1915998_1916762 | | -1.2 | 2.3E-03 |
| succinate dehydrogenase / fumarate reductase cytochrome b subunit | Ga0436255_01_4254379_4254765 | | -1.2 | 2.2E-02 |
| nucleoid-associated protein YgaU | Ga0436255_01_2716335_2716775 | | -1.2 | 8.2E-03 |
| capsular exopolysaccharide synthesis family protein | Ga0436255_01_4871871_4872677* | | -1.1 | 1.4E-02 |
| glutamate/aspartate transport system substrate-binding protein | Ga0436255_01_1227546_1228463 | | -1.1 | 3.2E-04 |
| DNA-binding protein HU-alpha | Ga0436255_01_2283538_2283837* | | -1.1 | 5.3E-03 |
| nitrogen regulatory protein P-II 2 | Ga0436255_01_2163491_2163829 | | -1.1 | 4.1E-03 |
| serine protein kinase | Ga0436255_01_2890307_2892229 | | -1.0 | 9.6E-03 |
| elongation factor G | Ga0436255_01_4406753_4408864 | | -0.9 | 8.7E-03 |
| 3-oxoadipyl-CoA thiolase | Ga0436255_01_1324437_1325639 | | -0.9 | 1.1E-02 |
| 2-isopropylmalate synthase | Ga0436255_01_3552675_3554348 | | -0.9 | 9.1E-03 |
| pilus assembly protein FimV | Ga0436255_01_782215_784887* | | -0.9 | 4.3E-02 |
| small subunit ribosomal protein S12 | Ga0436255_01_2953505_2953876 | | -0.8 | 5.7E-04 |
| protocatechuate 3,4-dioxygenase alpha subunit | Ga0436255_01_1557891_1558496 | | -0.8 | 8.7E-03 |
| 3-oxoadipate CoA-transferase beta subunit | Ga0436255_01_5391713_5392354 | | -0.8 | 6.1E-03 |
| isocitrate dehydrogenase | Ga0436255_01_4374278_4375534 | | -0.8 | 2.1E-02 |
| seryl-tRNA synthetase | Ga0436255_01_4385710_4386990 | | -0.7 | 5.9E-03 |
| carbonic anhydrase | Ga0436255_01_2327523_2328242 | | -0.6 | 5.7E-03 |
| glucose-6-phosphate isomerase | Ga0436255_01_1616958_1618703 | | -0.5 | 2.4E-02 |
| putrescine transport system substrate-binding protein | Ga0436255_01_2117751_2118848 | | -0.5 | 6.1E-03 |
| starvation-inducible DNA-binding protein | | Ga0436255_01_3742432_3742905 | -0.5 | 3.1E-03 |

| **Table S9.** Significantly (p ≤ 0.05) lower abundance proteins in *P. putida* M2 proteome in media containing glucose + ferulate with corresponding log_2_ fold change when compared with that in ferulate only. Only proteins with log_2_ FC ≥ 0.5 value are shown. | | | |
| --- | --- | --- | --- |
| **Protein name** | **Locus_tag** | **Log_2_ FC** | **p-value** |
| Ga0436255_01_3043696_3044832* | Ga0436255_01_3043696_3044832* | -3.1 | 3.2E-03 |
| acyl-CoA reductase-like NAD-dependent aldehyde dehydrogenase | Ga0436255_01_265990_267438 | -3.1 | 3.0E-03 |
| 3-oxoadipate enol-lactonase | Ga0436255_01_1320794_1321585 | -3.0 | 2.1E-02 |
| S-formylglutathione hydrolase | Ga0436255_01_3704270_3705124 | -2.7 | 8.5E-03 |
| feruloyl-CoA synthase | Ga0436255_01_264030_265913* | -2.6 | 4.1E-05 |
| 3-oxoadipate CoA-transferase alpha subunit | Ga0436255_01_5391009_5391704 | -2.6 | 6.4E-03 |
| 3-oxoadipyl-CoA thiolase | Ga0436255_01_1324437_1325639* | -2.5 | 5.0E-05 |
| Ga0436255_01_258380_260050* | Ga0436255_01_258380_260050* | -2.5 | 7.0E-03 |
| Ga0436255_01_4401307_4402632* | Ga0436255_01_4401307_4402632* | -2.5 | 3.1E-04 |
| malate synthase | Ga0436255_01_2838056_2840233 | -2.4 | 3.3E-04 |
| catalase-peroxidase | Ga0436255_01_49281_51536 | -2.4 | 2.3E-03 |
| 3-hydroxyacyl-CoA dehydrogenase/enoyl-CoA hydratase/3-hydroxybutyryl-CoA epimerase/enoyl-CoA isomerase | Ga0436255_01_655755_657902 | -2.3 | 1.1E-04 |
| vanillate O-demethylase monooxygenase subunit | Ga0436255_01_269163_270230* | -2.3 | 1.0E-02 |
| Ga0436255_01_1033189_1034253* | Ga0436255_01_1033189_1034253* | -2.3 | 2.0E-03 |
| Ga0436255_01_256740_258131* | Ga0436255_01_256740_258131* | -2.2 | 1.1E-04 |
| S-(hydroxymethyl)glutathione dehydrogenase/alcohol dehydrogenase | Ga0436255_01_3703149_3704261 | -2.2 | 3.0E-04 |
| Ga0436255_01_1443964_1444992* | Ga0436255_01_1443964_1444992* | -2.2 | 4.6E-03 |
| p-hydroxybenzoate 3-monooxygenase | Ga0436255_01_348086_349273* | -2.2 | 1.2E-03 |
| acyl dehydratase | Ga0436255_01_1753544_1753999 | -2.0 | 6.6E-03 |
| imipenem/basic amino acid-specific outer membrane pore | Ga0436255_01_3738846_3740129 | -2.0 | 3.5E-03 |
| Ga0436255_01_5414412_5415977 | Ga0436255_01_5414412_5415977 | -2.0 | 4.9E-03 |
| trans-feruloyl-CoA hydratase/vanillin synthase | Ga0436255_01_267514_268344 | -1.9 | 1.1E-04 |
| Ga0436255_01_1671621_1673171 | Ga0436255_01_1671621_1673171 | -1.8 | 1.4E-02 |
| protocatechuate 3,4-dioxygenase beta subunit | Ga0436255_01_1558507_1559226* | -1.7 | 4.0E-03 |
| Ga0436255_01_261038_262777* | Ga0436255_01_261038_262777* | -1.7 | 1.3E-02 |
| aldehyde dehydrogenase | Ga0436255_01_1761115_1762635 | -1.6 | 1.4E-03 |
| protocatechuate 3,4-dioxygenase alpha subunit | Ga0436255_01_1557891_1558496* | -1.6 | 8.6E-03 |
| Ga0436255_01_5287963_5289072* | Ga0436255_01_5287963_5289072* | -1.6 | 5.9E-03 |
| pilus assembly protein FimV | Ga0436255_01_782215_784887 | -1.5 | 4.5E-03 |
| branched-chain amino acid transport system substrate-binding protein | Ga0436255_01_1169501_1170616 | -1.5 | 2.8E-03 |
| Ga0436255_01_52955_53779* | Ga0436255_01_52955_53779* | -1.5 | 6.2E-04 |
| Ga0436255_01_761820_762770* | Ga0436255_01_761820_762770* | -1.5 | 8.1E-04 |
| Ga0436255_01_2744051_2744803* | Ga0436255_01_2744051_2744803* | -1.4 | 7.9E-04 |
| Ga0436255_01_559450_560928 [22] | Ga0436255_01_559450_560928 [22] | -1.4 | 7.1E-04 |
| putrescine transport system substrate-binding protein | Ga0436255_01_2117751_2118848 | -1.2 | 3.7E-03 |
| Ga0436255_01_2283538_2283837* | Ga0436255_01_2283538_2283837* | -1.2 | 2.1E-03 |
| Cu/Ag efflux protein CusF | Ga0436255_01_4975106_4975684 | -1.2 | 9.1E-03 |
| seryl-tRNA synthetase | Ga0436255_01_4385710_4386990 | -0.9 | 2.6E-02 |
| arginine/ornithine transport system substrate-binding protein | Ga0436255_01_3943313_3944098 | -0.9 | 6.1E-03 |
| hypothetical protein | Ga0436255_01_3301709_3303073 | -0.9 | 4.2E-03 |
| Ga0436255_01_5407420_5408097 | Ga0436255_01_5407420_5408097 | -0.9 | 1.6E-02 |
| Ga0436255_01_2672739_2674181 [2] | Ga0436255_01_2672739_2674181 [2] | -0.8 | 2.0E-02 |
| phosphonate transport system substrate-binding protein | Ga0436255_01_3381216_3382070 | -0.8 | 2.8E-02 |
| Ga0436255_01_4777849_4778391* | Ga0436255_01_4777849_4778391* | -0.8 | 2.3E-02 |
| hypothetical protein | Ga0436255_01_4643848_4644516 | -0.8 | 1.9E-02 |
| RHH-type proline utilization regulon transcriptional repressor/proline dehydrogenase/delta 1-pyrroline-5-carboxylate dehydrogenase | Ga0436255_01_1840552_1844505 | -0.7 | 4.4E-02 |
| nicotinamidase-related amidase | Ga0436255_01_3254337_3254957 | -0.7 | 7.9E-03 |
| Ga0436255_01_262792_264033* | Ga0436255_01_262792_264033* | -0.7 | 1.9E-02 |
| adhesin transport system outer membrane protein | Ga0436255_01_3898785_3900158 | -0.6 | 2.5E-02 |
| aconitate hydratase 2/2-methylisocitrate dehydratase | Ga0436255_01_527954_530578 | -0.6 | 3.1E-04 |
| glutamate/aspartate transport system substrate-binding protein | Ga0436255_01_1227546_1228463 | -0.6 | 1.8E-02 |
| poly(hydroxyalkanoate) granule-associated protein | Ga0436255_01_1915998_1916762 | -0.6 | 3.5E-02 |
| cystine transport system substrate-binding protein | Ga0436255_01_2682915_2683709 | -0.6 | 3.6E-02 |

| **Table S10.** Significantly (p ≤ 0.05) lower abundance proteins in *P. putida* M2 proteome in media containing glucose + vanillate with corresponding log_2_ fold change when compared with that in vanillate only. Only proteins with log_2_ FC ≥ 0.5 value are shown. | | | |
| --- | --- | --- | --- |
| **Protein name** | **Locus_tag** | **Log_2_ FC** | **p-value** |
| protocatechuate 3,4-dioxygenase beta subunit | Ga0436255_01_1558507_1559226 | -3.9 | 9.5E-03 |
| Ga0436255_01_5414412_5415977* | Ga0436255_01_5414412_5415977* | -3.1 | 7.2E-04 |
| catalase-peroxidase | Ga0436255_01_49281_51536 | -3.0 | 8.0E-03 |
| vanillate O-demethylase monooxygenase subunit | Ga0436255_01_269163_270230* | -2.8 | 1.2E-03 |
| aldehyde dehydrogenase | Ga0436255_01_1761115_1762635 | -2.7 | 2.7E-06 |
| 3-oxoadipate CoA-transferase alpha subunit | Ga0436255_01_5391009_5391704 | -2.6 | 5.9E-03 |
| isocitrate lyase | Ga0436255_01_4401307_4402632 | -2.5 | 1.7E-02 |
| Ga0436255_01_559450_560928 [22] | Ga0436255_01_559450_560928 [22] | -2.4 | 9.4E-03 |
| Ga0436255_01_1443964_1444992* | Ga0436255_01_1443964_1444992* | -2.4 | 3.9E-04 |
| outer membrane protein OmpA-like peptidoglycan-associated protein | Ga0436255_01_1186969_1187661 | -2.2 | 1.2E-04 |
| putrescine transport system substrate-binding protein | Ga0436255_01_2116496_2117542 | -2.2 | 2.8E-02 |
| protocatechuate 3,4-dioxygenase alpha subunit | Ga0436255_01_1557891_1558496* | -2.1 | 2.0E-04 |
| Ga0436255_01_575512_577251* | Ga0436255_01_575512_577251* | -2.1 | 8.5E-05 |
| Ga0436255_01_3043696_3044832* | Ga0436255_01_3043696_3044832* | -2.1 | 7.4E-03 |
| Ga0436255_01_1033189_1034253* | Ga0436255_01_1033189_1034253* | -2.1 | 3.7E-03 |
| S-formylglutathione hydrolase | Ga0436255_01_3704270_3705124 | -2.0 | 3.6E-03 |
| Ga0436255_01_4966930_4969179* | Ga0436255_01_4966930_4969179* | -2.0 | 2.8E-03 |
| Ga0436255_01_3738846_3740129* | Ga0436255_01_3738846_3740129* | -2.0 | 3.4E-03 |
| Cu/Ag efflux protein CusF | Ga0436255_01_4975106_4975684 | -1.9 | 1.2E-02 |
| Ga0436255_01_1671621_1673171 | Ga0436255_01_1671621_1673171 | -1.8 | 1.6E-02 |
| 3-oxoadipyl-CoA thiolase | Ga0436255_01_1324437_1325639* | -1.8 | 2.3E-03 |
| elongation factor G | Ga0436255_01_4406753_4408864 | -1.8 | 3.4E-03 |
| nicotinamidase-related amidase | Ga0436255_01_3254337_3254957 | -1.7 | 2.6E-03 |
| Ga0436255_01_1753544_1753999* | Ga0436255_01_1753544_1753999* | -1.6 | 3.6E-02 |
| Ga0436255_01_782215_784887* | Ga0436255_01_782215_784887* | -1.6 | 6.7E-03 |
| Ga0436255_01_2283538_2283837* | Ga0436255_01_2283538_2283837* | -1.6 | 5.4E-04 |
| p-hydroxybenzoate 3-monooxygenase | Ga0436255_01_348086_349273* | -1.6 | 7.2E-04 |
| Ga0436255_01_5407420_5408097 | Ga0436255_01_5407420_5408097 | -1.5 | 2.6E-02 |
| quinoprotein glucose dehydrogenase | Ga0436255_01_1253878_1256289 | -1.5 | 1.2E-02 |
| malate synthase | Ga0436255_01_2838056_2840233 | -1.5 | 7.0E-03 |
| Ga0436255_01_4871871_4872677 | Ga0436255_01_4871871_4872677 | -1.5 | 3.9E-05 |
| branched-chain amino acid transport system substrate-binding protein | Ga0436255_01_1169501_1170616 | -1.5 | 4.9E-04 |
| S-(hydroxymethyl)glutathione dehydrogenase/alcohol dehydrogenase | Ga0436255_01_3703149_3704261 | -1.5 | 7.7E-04 |
| 3-carboxy-cis,cis-muconate cycloisomerase | Ga0436255_01_1321597_1322949* | -1.5 | 2.6E-04 |
| cystine transport system substrate-binding protein | Ga0436255_01_2682915_2683709 | -1.4 | 1.3E-03 |
| Ga0436255_01_761820_762770* | Ga0436255_01_761820_762770* | -1.3 | 2.6E-03 |
| RHH-type proline utilization regulon transcriptional repressor/proline dehydrogenase/delta 1-pyrroline-5-carboxylate dehydrogenase | Ga0436255_01_1840552_1844505 | -1.3 | 9.2E-03 |
| hypothetical protein | Ga0436255_01_217567_217926 | -1.1 | 5.5E-03 |
| 3-hydroxyacyl-CoA dehydrogenase/enoyl-CoA hydratase/3-hydroxybutyryl-CoA epimerase/enoyl-CoA isomerase | Ga0436255_01_655755_657902 | -1.1 | 1.7E-02 |
| nucleoid-associated protein YgaU | Ga0436255_01_2716335_2716775 | -1.0 | 2.2E-03 |
| hypothetical protein | Ga0436255_01_3301709_3303073 | -1.0 | 3.2E-03 |
| adhesin transport system outer membrane protein | Ga0436255_01_3898785_3900158 | -0.9 | 1.8E-03 |
| Ga0436255_01_52955_53779* | Ga0436255_01_52955_53779* | -0.9 | 1.1E-03 |
| carbonic anhydrase | Ga0436255_01_2327523_2328242 | -0.9 | 2.8E-02 |
| cell division protein FtsA | Ga0436255_01_1398359_1399615 | -0.9 | 3.9E-02 |
| isocitrate dehydrogenase | Ga0436255_01_4374278_4375534 | -0.9 | 1.9E-02 |
| leucyl aminopeptidase | Ga0436255_01_3500925_3502418 | -0.8 | 3.0E-02 |
| purine-binding chemotaxis protein CheW | Ga0436255_01_4072011_4072490 | -0.8 | 1.3E-03 |
| Ga0436255_01_5287963_5289072* | Ga0436255_01_5287963_5289072* | -0.8 | 8.8E-03 |
| cysteine desulfurase | Ga0436255_01_3397480_3398694 | -0.8 | 1.0E-02 |
| glutamate/aspartate transport system substrate-binding protein | Ga0436255_01_1227546_1228463 | -0.7 | 1.1E-02 |
| 2,4-dienoyl-CoA reductase-like NADH-dependent reductase (Old Yellow Enzyme family) | Ga0436255_01_3619914_3621020 | -0.7 | 3.1E-02 |
| ATP-dependent RNA helicase DeaD | Ga0436255_01_881887_883566 | -0.7 | 2.5E-02 |
| hypothetical protein | Ga0436255_01_4643848_4644516 | -0.6 | 9.2E-03 |
| polyisoprenoid-binding protein YceI | Ga0436255_01_1886069_1886647 | -0.6 | 4.5E-02 |
| Ga0436255_01_549712_549963 | Ga0436255_01_549712_549963 | -0.5 | 4.6E-02 |
| D-alanine-D-alanine ligase | Ga0436255_01_1400506_1401462 | -0.5 | 4.9E-02 |

| **Table S11.** Significantly (p ≤ 0.05) lower abundance proteins in *P. putida* M2 proteome in media containing glucose + *p*-coumarate with corresponding log_2_ fold change when compared with that in glucose only. Only proteins with log_2_ FC ≥ 0.5 value are shown. | | | |
| --- | --- | --- | --- |
| **Protein name** | **Locus_tag** | **Log_2_ FC** | **p-value** |
| azurin | Ga0436255_01_3040630_3041076 | -2.2 | 4.3E-03 |
| O-succinylhomoserine sulfhydrylase | Ga0436255_01_774063_775274 | -2.1 | 8.7E-04 |
| starch phosphorylase | Ga0436255_01_1956339_1958789 | -1.7 | 1.4E-02 |
| 3-hydroxyacyl-CoA dehydrogenase/enoyl-CoA hydratase/3-hydroxybutyryl-CoA epimerase/enoyl-CoA isomerase | Ga0436255_01_655755_657902 | -1.4 | 1.2E-02 |
| glutamate dehydrogenase | Ga0436255_01_719906_724771 | -1.3 | 1.5E-02 |
| hypothetical protein | Ga0436255_01_1707707_1708459 | -1.2 | 2.1E-02 |
| aconitate hydratase | Ga0436255_01_680706_683447 | -1.1 | 7.7E-03 |
| branched-chain amino acid transport system substrate-binding protein | Ga0436255_01_1169501_1170616 | -1.0 | 3.2E-02 |
| glutaminyl-tRNA synthetase | Ga0436255_01_5629503_5631206 | -1.0 | 1.3E-02 |
| isocitrate dehydrogenase | Ga0436255_01_4374278_4375534 | -0.8 | 3.1E-03 |
| arginine/ornithine transport system substrate-binding protein | Ga0436255_01_3943313_3944098 | -0.8 | 3.1E-02 |
| 2-keto-3-deoxy-6-phosphogluconate aldolase | Ga0436255_01_3544702_3545376 | -0.8 | 1.0E-02 |
| ornithine decarboxylase | Ga0436255_01_3420662_3421825 | -0.8 | 5.0E-03 |
| aminopeptidase N | Ga0436255_01_748794_751451 | -0.7 | 7.4E-04 |
| large subunit ribosomal protein L32 | Ga0436255_01_819367_819549 | -0.7 | 2.8E-02 |
| elongation factor G | Ga0436255_01_4406753_4408864 | -0.6 | 2.5E-02 |
| glutamine synthetase | Ga0436255_01_1966094_1967500 | -0.6 | 9.8E-04 |
| NAD+ synthase | Ga0436255_01_3041191_3042018 | -0.6 | 3.6E-02 |
| hypothetical protein | Ga0436255_01_4738834_4739514* | -0.5 | 4.0E-02 |
| ornithine carbamoyltransferase | Ga0436255_01_3515593_3516603 | -0.5 | 1.7E-02 |

| **Table S12.** Significantly (p ≤ 0.05) lower abundance proteins in *P. putida* M2 proteome in media containing glucose + 4-hydroxybenzoate with corresponding log_2_ fold change when compared with that in glucose only. Only proteins with log_2_ FC ≥ 0.5 value are shown. | | | |
| --- | --- | --- | --- |
| **Protein name** | **Locus_tag** | **Log_2_ FC** | **p-value** |
| catalase-peroxidase | Ga0436255_01_49281_51536 | -1.2 | 3.1E-02 |
| nicotinamidase-related amidase | Ga0436255_01_3254337_3254957* | -1.2 | 3.5E-02 |
| branched-chain amino acid transport system substrate-binding protein | Ga0436255_01_1169501_1170616 | -1.0 | 3.5E-02 |
| aconitate hydratase | Ga0436255_01_680706_683447* | -1.0 | 4.7E-02 |
| 3-hydroxyacyl-CoA dehydrogenase/enoyl-CoA hydratase/3-hydroxybutyryl-CoA epimerase/enoyl-CoA isomerase | Ga0436255_01_655755_657902 | -1.0 | 2.0E-02 |
| 2,4-dienoyl-CoA reductase-like NADH-dependent reductase (Old Yellow Enzyme family) | Ga0436255_01_3619914_3621020 | -0.9 | 4.3E-02 |
| glutamine synthetase | Ga0436255_01_1966094_1967500 | -0.8 | 2.4E-03 |
| hypothetical protein | Ga0436255_01_2323954_2324595 | -0.8 | 1.4E-02 |
| nitrogen regulatory protein P-II 2 | Ga0436255_01_2163491_2163829 | -0.8 | 3.1E-02 |
| glutamate dehydrogenase | Ga0436255_01_719906_724771 | -0.7 | 2.1E-02 |
| elongation factor G | Ga0436255_01_4406753_4408864 | -0.7 | 8.1E-03 |
| isocitrate dehydrogenase | Ga0436255_01_4374278_4375534 | -0.7 | 9.8E-03 |
| ornithine carbamoyltransferase | Ga0436255_01_3515593_3516603 | -0.6 | 4.2E-02 |

| **Table S13.** Significantly (p ≤ 0.05) lower abundance proteins in *P. putida* M2 proteome in media containing glucose + ferulate with corresponding log_2_ fold change when compared with that in glucose only. Only proteins with log_2_ FC ≥ 0.5 value are shown. | | | |
| --- | --- | --- | --- |
| **Protein name** | **Locus_tag** | **Log_2_ FC** | **p-value** |
| large subunit ribosomal protein L9 | Ga0436255_01_3032202_3032648 | -0.9 | 1.6E-02 |
| nitroreductase/dihydropteridine reductase | Ga0436255_01_4677131_4677784 | -0.8 | 1.0E-02 |
| arginine/ornithine transport system substrate-binding protein | Ga0436255_01_3943313_3944098 | -0.8 | 2.2E-02 |
| glutamate synthase (NADPH/NADH) small chain | Ga0436255_01_2002698_2004116 | -0.6 | 2.9E-02 |
| cell division protein FtsZ | Ga0436255_01_1397107_1398303 | -0.6 | 2.9E-03 |
| enoyl-[acyl-carrier protein] reductase/trans-2-enoyl-CoA reductase (NAD+) | Ga0436255_01_1533218_1534417 | -0.5 | 3.3E-02 |

| **Table S14.** Significantly (p ≤ 0.05) lower abundance proteins in *P. putida* M2 proteome in media containing glucose + vanillate with corresponding log_2_ fold change when compared with that in glucose only. Only proteins with log_2_ FC ≥ 0.5 value are shown. | | | |
| --- | --- | --- | --- |
| **Protein name** | **Locus_tag** | **Log_2_ FC** | **p-value** |
| nitroreductase/dihydropteridine reductase | Ga0436255_01_4677131_4677784 | -1.1 | 8.1E-03 |
| arginine/ornithine transport system substrate-binding protein | Ga0436255_01_3943313_3944098 | -0.9 | 1.6E-02 |
| copper(I)-binding protein | Ga0436255_01_3076767_3077246 | -0.8 | 2.6E-02 |
| cell division protein FtsA | Ga0436255_01_1398359_1399615 | -0.8 | 4.6E-02 |
| cystine transport system substrate-binding protein | Ga0436255_01_2682915_2683709 | -0.7 | 3.3E-02 |
| cell division protein FtsZ | Ga0436255_01_1397107_1398303 | -0.6 | 1.7E-03 |
| citrate synthase | Ga0436255_01_4252737_4254026 | -0.6 | 4.5E-02 |
| serine protease Do | Ga0436255_01_1267340_1268779 | -0.6 | 2.8E-02 |

| **Table S15.** Significantly (p ≤ 0.05) lower abundance proteins in *P. putida* M2 proteome in media containing xylose + *p*-coumarate with corresponding log_2_ fold change when compared with that in *p*-coumarate only. Only proteins with log_2_ FC ≥ 0.5 value are shown. | | | |
| --- | --- | --- | --- |
| Protein name | Locus_tag | Log_2_ FC | p-value |
| arylsulfatase | Ga0436255_01_258380_260050 | -2.2 | 9.89E-04 |
| DNA-binding protein HU-alpha | Ga0436255_01_2283538_2283837 | -1.2 | 2.68E-03 |
| 4-carboxymuconolactone decarboxylase | Ga0436255_01_1320385_1320783 | -1.1 | 3.54E-02 |
| methyl-accepting chemotaxis protein | Ga0436255_01_559450_560928 [24] | -1.0 | 1.67E-02 |
| pyruvate carboxylase subunit B | Ga0436255_01_2523387_2525195 | -1.0 | 7.44E-03 |
| 3-oxoadipate CoA-transferase alpha subunit | Ga0436255_01_5391009_5391704 | -0.8 | 8.06E-03 |
| isocitrate lyase | Ga0436255_01_4401307_4402632 | -0.8 | 6.24E-05 |
| glutamate N-acetyltransferase/amino-acid N-acetyltransferase | Ga0436255_01_1391100_1392317 | -0.8 | 1.10E-02 |
| malate dehydrogenase (quinone) | Ga0436255_01_3287522_3289027 | -0.8 | 4.09E-02 |
| uroporphyrin-3 C-methyltransferase | Ga0436255_01_2145309_2146409 | -0.7 | 4.13E-02 |
| alkylation response protein AidB-like acyl-CoA dehydrogenase | Ga0436255_01_261038_262777 | -0.7 | 5.61E-03 |
| glutamate/aspartate transport system substrate-binding protein | Ga0436255_01_1227546_1228463 | -0.7 | 3.18E-02 |
| threonine synthase | Ga0436255_01_3611765_3613174 | -0.7 | 4.08E-02 |
| 3-oxoadipate enol-lactonase | Ga0436255_01_1320794_1321585 | -0.7 | 9.38E-04 |
| feruloyl-CoA synthase | Ga0436255_01_264030_265913 | -0.7 | 2.27E-03 |
| 3-carboxy-cis,cis-muconate cycloisomerase | Ga0436255_01_1321597_1322949 | -0.7 | 2.36E-02 |
| nicotinamidase-related amidase | Ga0436255_01_3254337_3254957 | -0.7 | 3.83E-03 |
| trans-feruloyl-CoA hydratase/vanillin synthase | Ga0436255_01_267514_268344 | -0.7 | 3.54E-02 |
| beta-alanine--pyruvate transaminase | Ga0436255_01_3143828_3145174 | -0.6 | 3.16E-02 |
| acetylornithine/N-succinyldiaminopimelate aminotransferase | Ga0436255_01_3947734_3948954 | -0.6 | 6.86E-03 |
| pyruvate carboxylase subunit A | Ga0436255_01_2521873_2523288 | -0.5 | 4.04E-02 |
| electron-transferring-flavoprotein dehydrogenase | Ga0436255_01_4243592_4245256 | -0.5 | 2.79E-02 |
| p-hydroxybenzoate 3-monooxygenase | Ga0436255_01_348086_349273 | -0.5 | 9.02E-03 |

| **Table S16.** Significantly (p ≤ 0.05) lower abundance proteins in *P. putida* M2 proteome in media containing xylose + 4-hydroxybenzoate with corresponding log_2_ fold change when compared with that in 4-hydroxybenzoate only. Only proteins with log_2_ FC ≥ 0.5 value are shown. | | | |
| --- | --- | --- | --- |
| **Protein name** | **Locus_tag** | **Log_2_ FC** | **p-value** |
| gamma-glutamyltranspeptidase/glutathione hydrolase | Ga0436255_01_1562537_1564204 | -2.4 | 7.6E-04 |
| pyruvate carboxylase subunit A | Ga0436255_01_2521873_2523288 | -1.9 | 4.0E-03 |
| pilus assembly protein FimV | Ga0436255_01_782215_784887 | -1.4 | 3.3E-02 |
| pyruvate carboxylase subunit B | Ga0436255_01_2523387_2525195 | -1.3 | 1.1E-02 |
| DNA-binding protein HU-alpha | Ga0436255_01_2283538_2283837 | -1.3 | 1.8E-03 |
| 2,4-dienoyl-CoA reductase-like NADH-dependent reductase (Old Yellow Enzyme family) | Ga0436255_01_3619914_3621020 | -1.2 | 1.6E-03 |
| spore cortex formation protein SpoVR/YcgB (stage V sporulation) | Ga0436255_01_2887294_2888862 | -1.2 | 1.7E-02 |
| 2,5-diketo-D-gluconate reductase B | Ga0436255_01_52955_53779 | -1.1 | 1.8E-02 |
| 4-carboxymuconolactone decarboxylase | Ga0436255_01_1320385_1320783 | -1.0 | 3.8E-03 |
| general L-amino acid transport system substrate-binding protein | Ga0436255_01_1443964_1444992 | -1.0 | 1.3E-02 |
| carbon starvation protein | Ga0436255_01_1541217_1543283 | -0.9 | 1.1E-03 |
| glutamate dehydrogenase | Ga0436255_01_719906_724771 | -0.9 | 1.3E-04 |
| 3-oxoadipate CoA-transferase alpha subunit | Ga0436255_01_5391009_5391704 | -0.8 | 4.7E-02 |
| Ga0436255_01_4643848_4644516 | Ga0436255_01_4643848_4644516 | -0.8 | 5.0E-02 |
| peptidyl-prolyl cis-trans isomerase B (cyclophilin B) | Ga0436255_01_5631456_5631956 | -0.8 | 2.3E-02 |
| peroxiredoxin (alkyl hydroperoxide reductase subunit C) | Ga0436255_01_4540982_4541545 | -0.8 | 2.3E-02 |
| serine protein kinase | Ga0436255_01_2890307_2892229 | -0.7 | 1.1E-02 |
| branched-chain amino acid transport system substrate-binding protein | Ga0436255_01_3043696_3044832 | -0.7 | 2.5E-02 |
| capsular exopolysaccharide synthesis family protein | Ga0436255_01_4871871_4872677 | -0.7 | 3.2E-02 |
| p-hydroxybenzoate 3-monooxygenase | Ga0436255_01_348086_349273 | -0.7 | 7.3E-03 |
| 3-oxoadipate enol-lactonase | Ga0436255_01_1320794_1321585 | -0.7 | 1.2E-03 |
| methyl-accepting chemotaxis protein | Ga0436255_01_559450_560928 [24] | -0.6 | 3.5E-03 |
| elongation factor G | Ga0436255_01_4406753_4408864 | -0.6 | 1.7E-03 |
| Ga0436255_01_5414412_5415977 | Ga0436255_01_5414412_5415977 | -0.6 | 7.1E-04 |
| pyruvate dehydrogenase E2 component (dihydrolipoamide acetyltransferase) | Ga0436255_01_2811549_2813192 | -0.6 | 7.9E-03 |
| isocitrate dehydrogenase | Ga0436255_01_4374278_4375534 | -0.6 | 2.1E-02 |
| purine-binding chemotaxis protein CheW | Ga0436255_01_4072011_4072490 | -0.5 | 7.9E-03 |
| large subunit ribosomal protein L17 | Ga0436255_01_2971293_2971679 | -0.5 | 3.5E-02 |
| nicotinamidase-related amidase | Ga0436255_01_3254337_3254957 | -0.5 | 1.2E-02 |
| branched-chain amino acid transport system substrate-binding protein | Ga0436255_01_1169501_1170616 | -0.5 | 2.7E-02 |

| **Table S17.** Significantly (p ≤ 0.05) lower abundance proteins in *P. putida* M2 proteome in media containing xylose + ferulate with corresponding log_2_ fold change when compared with that in ferulate only. Only proteins with log_2_ FC ≥ 0.5 value are shown. | | | |
| --- | --- | --- | --- |
| **Protein name** | **Locus_tag** | **Log_2_ FC** | **p-value** |
| isoquinoline 1-oxidoreductase beta subunit | Ga0436255_01_4966930_4969179 | -2.9 | 5.5E-06 |
| pyruvate carboxylase subunit B | Ga0436255_01_2523387_2525195 | -2.7 | 4.4E-02 |
| 4-carboxymuconolactone decarboxylase | Ga0436255_01_1320385_1320783 | -2.2 | 4.9E-03 |
| TRAP transporter TAXI family solute receptor | Ga0436255_01_761820_762770 | -2.0 | 7.7E-03 |
| glycine betaine/proline transport system substrate-binding protein | Ga0436255_01_2759427_2760374 | -2.0 | 1.2E-02 |
| quinate dehydrogenase (quinone) | Ga0436255_01_4827183_4829600 | -1.9 | 3.8E-03 |
| 3-carboxy-cis,cis-muconate cycloisomerase | Ga0436255_01_1321597_1322949 | -1.9 | 4.7E-03 |
| p-hydroxybenzoate 3-monooxygenase | Ga0436255_01_348086_349273 | -1.9 | 9.7E-03 |
| arylsulfatase | Ga0436255_01_258380_260050 | -1.8 | 3.0E-04 |
| acetyl-CoA C-acetyltransferase | Ga0436255_01_262792_264033 | -1.8 | 1.9E-02 |
| hypothetical protein | Ga0436255_01_3987214_3987936 | -1.8 | 2.1E-02 |
| general L-amino acid transport system substrate-binding protein | Ga0436255_01_1443964_1444992 | -1.8 | 3.0E-05 |
| glycerol transport system substrate-binding protein | Ga0436255_01_575512_577251* | -1.8 | 1.1E-03 |
| 2,5-diketo-D-gluconate reductase B | Ga0436255_01_52955_53779 | -1.8 | 1.8E-02 |
| universal stress protein A | Ga0436255_01_658935_659375 | -1.7 | 6.2E-03 |
| starch phosphorylase | Ga0436255_01_1956339_1958789 | -1.7 | 5.5E-03 |
| pilus assembly protein FimV | Ga0436255_01_782215_784887 | -1.7 | 1.8E-02 |
| branched-chain amino acid transport system substrate-binding protein | Ga0436255_01_3043696_3044832 | -1.6 | 7.4E-05 |
| methyl-accepting chemotaxis protein | Ga0436255_01_559450_560928 [24] | -1.5 | 2.1E-02 |
| outer membrane PBP1 activator LpoA protein | Ga0436255_01_1415696_1417513 | -1.5 | 3.1E-02 |
| pyruvate carboxylase subunit A | Ga0436255_01_2521873_2523288* | -1.4 | 1.9E-02 |
| 3-oxoadipate enol-lactonase | Ga0436255_01_1320794_1321585 | -1.2 | 2.1E-03 |
| RHH-type proline utilization regulon transcriptional repressor/proline dehydrogenase/delta 1-pyrroline-5-carboxylate dehydrogenase | Ga0436255_01_1840552_1844505 [2] | -1.2 | 3.3E-02 |
| 3-oxoadipate CoA-transferase beta subunit | Ga0436255_01_5391713_5392354* | -1.2 | 4.0E-02 |
| outer membrane protein OmpA-like peptidoglycan-associated protein | Ga0436255_01_1186969_1187661 | -1.1 | 4.5E-02 |
| hypothetical protein | Ga0436255_01_217567_217926 | -1.1 | 3.9E-02 |
| 3-oxoadipate CoA-transferase alpha subunit | Ga0436255_01_5391009_5391704 | -1.1 | 8.0E-03 |
| quinoprotein glucose dehydrogenase | Ga0436255_01_1253878_1256289 | -1.0 | 3.4E-03 |
| S-formylglutathione hydrolase | Ga0436255_01_3704270_3705124 | -1.0 | 1.4E-03 |
| putrescine transport system substrate-binding protein | Ga0436255_01_2116496_2117542 | -1.0 | 7.1E-03 |
| malonate-semialdehyde dehydrogenase (acetylating)/methylmalonate-semialdehyde dehydrogenase | Ga0436255_01_1569586_1571112* | -0.9 | 4.2E-02 |
| isocitrate dehydrogenase | Ga0436255_01_4374278_4375534 | -0.9 | 1.8E-02 |
| branched-chain amino acid transport system substrate-binding protein | Ga0436255_01_1169501_1170616 | -0.9 | 1.2E-02 |
| trans-feruloyl-CoA hydratase/vanillin synthase | Ga0436255_01_267514_268344 | -0.8 | 8.9E-04 |
| capsular exopolysaccharide synthesis family protein | Ga0436255_01_4871871_4872677 | -0.7 | 1.2E-02 |
| alkylation response protein AidB-like acyl-CoA dehydrogenase | Ga0436255_01_261038_262777 | -0.7 | 3.5E-02 |
| glutamate/aspartate transport system substrate-binding protein | Ga0436255_01_1227546_1228463 | -0.7 | 8.5E-03 |
| S-(hydroxymethyl)glutathione dehydrogenase/alcohol dehydrogenase | Ga0436255_01_3703149_3704261 | -0.7 | 4.4E-03 |
| ubiquinol-cytochrome c reductase cytochrome c1 subunit | Ga0436255_01_1420675_1421454 | -0.7 | 2.6E-02 |
| catalase-peroxidase | Ga0436255_01_49281_51536 | -0.7 | 1.9E-03 |
| cystine transport system substrate-binding protein | Ga0436255_01_2682915_2683709 | -0.7 | 3.8E-02 |
| DNA-binding protein HU-alpha | Ga0436255_01_2283538_2283837 | -0.7 | 9.4E-03 |
| 3-oxoadipyl-CoA thiolase | Ga0436255_01_1324437_1325639 | -0.7 | 8.4E-05 |
| hypothetical protein | Ga0436255_01_4777849_4778391 | -0.6 | 1.9E-03 |
| aldehyde dehydrogenase | Ga0436255_01_1761115_1762635 [2] | -0.6 | 1.1E-02 |
| polar amino acid transport system substrate-binding protein | Ga0436255_01_2744051_2744803 | -0.6 | 1.3E-02 |
| arginyl-tRNA synthetase | Ga0436255_01_2023730_2025466 | -0.6 | 3.5E-02 |
| acyl-CoA reductase-like NAD-dependent aldehyde dehydrogenase | Ga0436255_01_265990_267438 | -0.5 | 2.2E-03 |
| S-(hydroxymethyl)glutathione dehydrogenase/alcohol dehydrogenase | Ga0436255_01_5287963_5289072* | -0.5 | 1.7E-02 |
| poly(hydroxyalkanoate) granule-associated protein | Ga0436255_01_1915998_1916762 | -0.5 | 7.8E-03 |
| uroporphyrin-3 C-methyltransferase | Ga0436255_01_2145309_2146409 | -0.5 | 8.5E-03 |
| citrate synthase | Ga0436255_01_4252737_4254026 | -0.5 | 2.3E-02 |
| carbon starvation protein | Ga0436255_01_1541217_1543283 | -0.5 | 1.1E-02 |

| **Table S18.** Significantly (p ≤ 0.05) lower abundance proteins in *P. putida* M2 proteome in media containing xylose + vanillate with corresponding log_2_ fold change when compared with that in vanillate only. Only proteins with log_2_ FC ≥ 0.5 value are shown. | | | |
| --- | --- | --- | --- |
| **Protein name** | **Locus_tag** | **Log_2_ FC** | **p-value** |
| gamma-glutamyltranspeptidase/glutathione hydrolase | Ga0436255_01_1562537_1564204 | -2.8 | 6.3E-04 |
| isoquinoline 1-oxidoreductase beta subunit | Ga0436255_01_4966930_4969179 | -2.4 | 8.6E-04 |
| protocatechuate 3,4-dioxygenase alpha subunit | Ga0436255_01_1557891_1558496 | -2.2 | 2.6E-03 |
| 3-oxoadipate CoA-transferase beta subunit | Ga0436255_01_5391713_5392354 | -2.0 | 3.8E-04 |
| general L-amino acid transport system substrate-binding protein | Ga0436255_01_1443964_1444992 | -1.8 | 3.1E-03 |
| 2,5-diketo-D-gluconate reductase B | Ga0436255_01_52955_53779 | -1.8 | 2.4E-04 |
| 4-carboxymuconolactone decarboxylase | Ga0436255_01_1320385_1320783 | -1.6 | 1.8E-03 |
| glycerol transport system substrate-binding protein | Ga0436255_01_575512_577251* | -1.6 | 8.6E-03 |
| pyruvate carboxylase subunit B | Ga0436255_01_2523387_2525195 | -1.5 | 4.0E-02 |
| hypothetical protein | Ga0436255_01_217567_217926 | -1.5 | 2.4E-02 |
| spore cortex formation protein SpoVR/YcgB (stage V sporulation) | Ga0436255_01_2887294_2888862 | -1.4 | 1.2E-02 |
| TRAP transporter TAXI family solute receptor | Ga0436255_01_761820_762770 | -1.4 | 9.7E-03 |
| polysaccharide export outer membrane protein | Ga0436255_01_4868571_4869128 | -1.4 | 2.1E-03 |
| 3-carboxy-cis,cis-muconate cycloisomerase | Ga0436255_01_1321597_1322949 | -1.3 | 5.4E-03 |
| putative thioredoxin | Ga0436255_01_1793863_1794735 | -1.3 | 2.9E-02 |
| 3-oxoadipate enol-lactonase | Ga0436255_01_1320794_1321585 | -1.3 | 1.0E-02 |
| putative NADH-flavin reductase | Ga0436255_01_1305839_1306453* | -1.2 | 4.9E-02 |
| quinate dehydrogenase (quinone) | Ga0436255_01_4827183_4829600 | -1.2 | 3.4E-02 |
| 3-oxoadipate CoA-transferase alpha subunit | Ga0436255_01_5391009_5391704 | -1.2 | 2.5E-04 |
| UDP-N-acetylmuramoyl-tripeptide--D-alanyl-D-alanine ligase | Ga0436255_01_1407615_1408982 | -1.0 | 5.2E-03 |
| S-formylglutathione hydrolase | Ga0436255_01_3704270_3705124 | -1.0 | 3.9E-03 |
| branched-chain amino acid transport system substrate-binding protein | Ga0436255_01_3043696_3044832 | -1.0 | 6.2E-03 |
| S-(hydroxymethyl)glutathione dehydrogenase/alcohol dehydrogenase | Ga0436255_01_5287963_5289072* | -1.0 | 6.0E-05 |
| glutamate/aspartate transport system substrate-binding protein | Ga0436255_01_1227546_1228463 | -0.9 | 5.8E-03 |
| starch phosphorylase | Ga0436255_01_1956339_1958789 | -0.8 | 3.8E-02 |
| malonate-semialdehyde dehydrogenase (acetylating)/methylmalonate-semialdehyde dehydrogenase | Ga0436255_01_1569586_1571112* | -0.8 | 3.2E-02 |
| elongation factor G | Ga0436255_01_4406753_4408864 | -0.8 | 1.7E-04 |
| RHH-type proline utilization regulon transcriptional repressor/proline dehydrogenase/delta 1-pyrroline-5-carboxylate dehydrogenase | Ga0436255_01_1840552_1844505 [2] | -0.8 | 2.6E-02 |
| glutamate dehydrogenase | Ga0436255_01_719906_724771 | -0.8 | 9.0E-03 |
| isocitrate dehydrogenase | Ga0436255_01_4374278_4375534 | -0.8 | 1.2E-03 |
| capsular exopolysaccharide synthesis family protein | Ga0436255_01_4871871_4872677 | -0.8 | 1.5E-02 |
| 2,4-dienoyl-CoA reductase-like NADH-dependent reductase (Old Yellow Enzyme family) | Ga0436255_01_3619914_3621020 | -0.7 | 1.2E-02 |
| cyclopropane-fatty-acyl-phospholipid synthase | Ga0436255_01_2502635_2503819 | -0.7 | 1.3E-02 |
| DNA gyrase subunit A | Ga0436255_01_976902_979673 | -0.7 | 5.0E-02 |
| S-(hydroxymethyl)glutathione dehydrogenase/alcohol dehydrogenase | Ga0436255_01_3703149_3704261 | -0.7 | 1.4E-02 |
| glucose-6-phosphate isomerase | Ga0436255_01_1616958_1618703 | -0.6 | 3.5E-02 |
| pyruvate dehydrogenase E2 component (dihydrolipoamide acetyltransferase) | Ga0436255_01_2811549_2813192 | -0.6 | 1.5E-02 |
| adhesin transport system outer membrane protein | Ga0436255_01_3898785_3900158 | -0.6 | 1.4E-03 |
| quinoprotein glucose dehydrogenase | Ga0436255_01_1253878_1256289 | -0.6 | 1.7E-02 |
| vanillate O-demethylase monooxygenase subunit | Ga0436255_01_269163_270230 | -0.6 | 3.1E-02 |
| ATP-dependent Clp protease protease subunit | Ga0436255_01_571022_571663 | -0.6 | 1.6E-02 |
| succinate dehydrogenase / fumarate reductase flavoprotein subunit | Ga0436255_01_4255131_4256903 | -0.6 | 2.9E-03 |

| **Table S19.** Significantly (p ≤ 0.05) lower abundance proteins in *P. putida* M2 proteome in media containing xylose + *p*-coumarate with corresponding log_2_ fold change when compared with that in xylose only. Only proteins with log_2_ FC ≥ 0.5 value are shown. | | | |
| --- | --- | --- | --- |
| **Protein name** | **Locus_tag** | **Log_2_ FC** | **p-value** |
| 5-methyltetrahydropteroyltriglutamate--homocysteine methyltransferase | Ga0436255_01_1535993_1538293 | -1.4 | 1.0E-04 |
| small subunit ribosomal protein S19 | Ga0436255_01_2960786_2961061 | -1.2 | 9.9E-03 |
| PhzF family phenazine biosynthesis protein | Ga0436255_01_4086380_4087168 | -1.1 | 2.6E-02 |
| DNA-binding protein HU-alpha | Ga0436255_01_2283538_2283837 | -1.1 | 3.9E-03 |
| glycogen operon protein | Ga0436255_01_480029_482182 | -0.9 | 3.4E-02 |
| hypothetical protein | Ga0436255_01_3987214_3987936 | -0.9 | 1.2E-02 |
| acetylglutamate kinase | Ga0436255_01_2261336_2262241 | -0.9 | 8.6E-04 |
| NADP-dependent aldehyde dehydrogenase | Ga0436255_01_4982951_4984531 | -0.8 | 1.6E-02 |
| methyl-accepting chemotaxis protein | Ga0436255_01_559450_560928 [24] | -0.8 | 4.8E-03 |
| multiple sugar transport system ATP-binding protein | Ga0436255_01_3537937_3539091 | -0.8 | 2.6E-03 |
| 2-isopropylmalate synthase | Ga0436255_01_3552675_3554348 | -0.7 | 5.4E-04 |
| 2-keto-3-deoxyxylonate dehydratase | Ga0436255_01_4615537_4616718 | -0.7 | 2.3E-04 |
| 3-hydroxyacyl-CoA dehydrogenase/enoyl-CoA hydratase/3-hydroxybutyryl-CoA epimerase/enoyl-CoA isomerase | Ga0436255_01_655755_657902 | -0.7 | 1.2E-02 |
| general L-amino acid transport system substrate-binding protein | Ga0436255_01_1443964_1444992 | -0.6 | 1.6E-02 |
| threonine synthase | Ga0436255_01_3611765_3613174 | -0.6 | 3.4E-02 |
| ATP-binding cassette ChvD family protein | Ga0436255_01_3212621_3214288 | -0.6 | 2.8E-03 |
| branched-chain amino acid transport system substrate-binding protein | Ga0436255_01_1169501_1170616 | -0.6 | 1.3E-02 |
| branched-chain amino acid transport system substrate-binding protein | Ga0436255_01_3043696_3044832 | -0.6 | 3.8E-02 |
| histidinol dehydrogenase | Ga0436255_01_3488894_3490219 | -0.5 | 2.4E-02 |
| glucose/mannose transport system substrate-binding protein | Ga0436255_01_3534707_3535993 | -0.5 | 1.1E-02 |

| **Table S20.** Significantly (p ≤ 0.05) lower abundance proteins in *P. putida* M2 proteome in media containing xylose + 4-hydroxybenzoate with corresponding log_2_ fold change when compared with that in xylose only. Only proteins with log_2_ FC ≥ 0.5 value are shown. | | | |
| --- | --- | --- | --- |
| **Protein name** | **Locus_tag** | **Log_2_ FC** | **p-value** |
| 5-methyltetrahydropteroyltriglutamate--homocysteine methyltransferase | Ga0436255_01_1535993_1538293 | -1.4 | 6.4E-05 |
| PhzF family phenazine biosynthesis protein | Ga0436255_01_4086380_4087168 | -1.3 | 3.6E-02 |
| 3-hydroxy acid dehydrogenase/malonic semialdehyde reductase | Ga0436255_01_1816117_1816878 | -0.9 | 1.5E-02 |
| NADP-dependent aldehyde dehydrogenase | Ga0436255_01_4982951_4984531 | -0.8 | 1.9E-02 |
| small subunit ribosomal protein S19 | Ga0436255_01_2960786_2961061 | -0.8 | 2.6E-02 |
| purine-binding chemotaxis protein CheW | Ga0436255_01_4072011_4072490 | -0.7 | 5.0E-02 |
| tryptophan synthase alpha chain | Ga0436255_01_2342737_2343546 | -0.7 | 4.5E-02 |
| acetylglutamate kinase | Ga0436255_01_2261336_2262241 | -0.7 | 9.7E-04 |
| 2-isopropylmalate synthase | Ga0436255_01_3552675_3554348 | -0.6 | 5.3E-03 |
| DNA-binding protein HU-alpha | Ga0436255_01_2283538_2283837 | -0.6 | 1.8E-02 |
| glycine hydroxymethyltransferase | Ga0436255_01_3206478_3207731 | -0.6 | 8.6E-05 |
| thioredoxin 1 | Ga0436255_01_2648598_2648927 | -0.6 | 4.3E-02 |
| TolB protein | Ga0436255_01_3751805_3753076 | -0.6 | 3.0E-02 |

| **Table S21.** Significantly (p ≤ 0.05) lower abundance proteins in *P. putida* M2 proteome in media containing xylose + ferulate with corresponding log_2_ fold change when compared with that in xylose only. Only proteins with log_2_ FC ≥ 0.5 value are shown. | | | |
| --- | --- | --- | --- |
| **Protein name** | **Locus_tag** | **Log_2_ FC** | **p-value** |
| hypothetical protein | Ga0436255_01_3987214_3987936 | -1.7 | 2.8E-03 |
| 5-methyltetrahydropteroyltriglutamate--homocysteine methyltransferase | Ga0436255_01_1535993_1538293 | -1.6 | 8.8E-04 |
| NADP-dependent aldehyde dehydrogenase | Ga0436255_01_4982951_4984531 | -0.9 | 1.7E-02 |
| 2-isopropylmalate synthase | Ga0436255_01_3552675_3554348 | -0.9 | 8.4E-04 |
| cytochrome c5 | Ga0436255_01_2241544_2241966 | -0.7 | 1.1E-02 |
| acetylglutamate kinase | Ga0436255_01_2261336_2262241 | -0.6 | 1.3E-02 |
| large subunit ribosomal protein L19 | Ga0436255_01_3605650_3606000 | -0.5 | 3.2E-02 |

| **Table S22.** Significantly (p ≤ 0.05) lower abundance proteins in *P. putida* M2 proteome in media containing xylose + vanillate with corresponding log_2_ fold change when compared with that in xylose only. Only proteins with log_2_ FC ≥ 0.5 value are shown. | | | |
| --- | --- | --- | --- |
| **Protein name** | **Locus_tag** | **Log_2_ FC** | **p-value** |
| 2-isopropylmalate synthase | Ga0436255_01_3552675_3554348 | -1.0 | 3.4E-04 |
| glutathione S-transferase | Ga0436255_01_2150873_2151535 | -0.7 | 3.1E-03 |
| threonine synthase | Ga0436255_01_3611765_3613174 | -0.7 | 1.9E-02 |
| DNA gyrase subunit A | Ga0436255_01_976902_979673 | -0.6 | 1.5E-02 |
| large subunit ribosomal protein L2 | Ga0436255_01_2959945_2960769 | -0.6 | 7.9E-03 |
| 5-methyltetrahydropteroyltriglutamate--homocysteine methyltransferase | Ga0436255_01_1535993_1538293 | -0.6 | 5.8E-03 |
| (R)-2,3-dihydroxy-3-methylpentanoate dehydratase | Ga0436255_01_2061066_2062907 | -0.5 | 2.3E-02 |
| nucleoside-diphosphate kinase | Ga0436255_01_3402574_3402999 | -0.5 | 2.9E-02 |

| **Table S23.** Significantly (p ≤ 0.05) lower abundance proteins in *Pseudomonas alloputida* KT2440 proteome in media containing glucose + *p*-coumarate with corresponding log_2_ fold change when compared with that in *p*-coumarate only. Only proteins with log_2_ FC ≥ 0.5 value are shown. | | | |
| --- | --- | --- | --- |
| **Protein name** | **Locus_tag** | **Log_2_ FC** | **p-value** |
| Dehydrogenase subunit, putative | PP_1661 | -3.5 | 8.7E-04 |
| Branched-chain amino acid ABC transporter, periplasmic amino acid-binding protein | PP_4867 | -3.2 | 8.0E-04 |
| Isocitrate lyase | aceA | -2.9 | 1.1E-02 |
| 2,4-dienoyl-CoA reductase FadH | fadH | -2.6 | 1.1E-03 |
| Uncharacterized protein | PP_1659 | -2.5 | 1.4E-03 |
| Arylsulfatase, putative | PP_3352 | -2.4 | 3.6E-03 |
| 3-carboxy-cis,cis-muconate cycloisomerase | pcaB | -2.0 | 2.1E-04 |
| Surface adhesion protein, putative | PP_0168 | -2.0 | 1.6E-02 |
| Branched-chain amino acid ABC transporter, periplasmic amino acid-binding protein | braC | -1.9 | 2.5E-03 |
| 4-hydroxybenzoate transporter | pcaK* | -1.8 | 2.1E-02 |
| Hydrolase, isochorismatase family | PP_0711 | -1.8 | 4.5E-03 |
| N-carbamoyl-beta-alanine amidohydrolase, putative | PP_4034 | -1.8 | 1.1E-02 |
| Uncharacterized protein | PP_3536 | -1.7 | 1.8E-03 |
| Malate synthase G | glcB | -1.7 | 5.0E-04 |
| Electron transfer flavoprotein-ubiquinone oxidoreductase, putative | PP_4203 | -1.7 | 3.4E-03 |
| OmpA family protein |  | -1.7 | 1.3E-02 |
| Cluster of Methyl-accepting chemotaxis transducer | PP_2310 | -1.6 | 8.1E-03 |
| Elongation factor G 2 | fusB | -1.6 | 3.9E-03 |
| Acetolactate synthase, catabolic, putative | PP_1157 | -1.6 | 2.1E-02 |
| Protocatechuate 3,4-dioxygenase, beta subunit | pcaH | -1.6 | 2.2E-03 |
| Uncharacterized protein | PP_3611 | -1.4 | 6.0E-03 |
| Uncharacterized protein | PP_2006 | -1.4 | 2.4E-04 |
| ABC transporter, periplasmic binding protein | PP_1726 | -1.4 | 2.9E-02 |
| 3-oxoadipate CoA-transferase subunit B | pcaJ | -1.4 | 4.3E-02 |
| Uncharacterized protein | PP_1993* | -1.3 | 2.8E-02 |
| 4-hydroxybenzoate hydroxylase | pobA | -1.3 | 7.4E-05 |
| Uncharacterized protein | PP_3145 | -1.3 | 7.1E-03 |
| Xenobiotic reductase, putative | PP_1478 | -1.2 | 1.0E-02 |
| Glutamate synthase, small subunit, putative | PP_4037 | -1.2 | 1.0E-03 |
| Polyhydroxyalkanoate granule-associated protein GA2 | PP_5007 | -1.1 | 4.9E-02 |
| Uncharacterized protein | PP_0766 | -1.1 | 2.6E-03 |
| Acyl-CoA dehydrogenase, ferrulic acid biotransformation protein, putative | PP_3354 | -1.1 | 5.8E-03 |
| Gamma-glutamyltransferase | ggt-2* | -1.1 | 1.8E-02 |
| Amino acid ABC transporter, periplasmic amino acid-binding protein | PP_1071 | -1.0 | 2.4E-02 |
| Vanillin dehydrogenase | vdh | -1.0 | 1.8E-02 |
| General amino acid ABC transporter, periplasmic binding protein | aapJ* | -1.0 | 1.6E-02 |
| Polyamine ABC transporter, periplasmic polyamine-binding protein | PP_1486* | -1.0 | 1.5E-02 |
| Feruloyl-CoA-synthetase | fcs | -1.0 | 7.9E-03 |
| DNA-binding protein HU-alpha | hupA* | -0.9 | 7.6E-04 |
| Tail-specific protease prc | prc | -0.9 | 2.6E-02 |
| Dipeptide ABC transporter, periplasmic peptide-binding protein | PP_0885* | -0.9 | 3.3E-02 |
| Uncharacterized protein | PP_2080 | -0.8 | 2.2E-03 |
| Surface adhesion protein, putative | PP_0806 | -0.8 | 3.7E-02 |
| DNA-binding stress protein, putative | PP_1210 | -0.8 | 8.6E-04 |
| 2-isopropylmalate synthase | leuA | -0.8 | 3.5E-03 |
| Beta-ketoadipyl CoA thiolase PcaF | pcaF | -0.8 | 2.4E-03 |
| Isocitrate dehydrogenase [NADP] | icd | -0.7 | 2.9E-02 |
| Uncharacterized protein | PP_0397 | -0.7 | 9.7E-03 |
| Protocatechuate 3,4-dioxygenase, alpha subunit | pcaG | -0.7 | 2.6E-02 |
| Carbonic anhydrase | cynT | -0.6 | 3.2E-02 |
| P-47-related protein | PP_2007* | -0.6 | 9.3E-03 |
| 5-aminovalerate aminotransferase DavT | davT | -0.6 | 2.0E-02 |
| D-alanyl-D-alanine carboxypeptidase | dacA | -0.6 | 1.8E-02 |
| PhoH family protein | PP_1291 | -0.6 | 1.5E-03 |
| 3-oxoadipate enol-lactone hydrolase | pcaD | -0.6 | 4.8E-03 |
| Carbamate kinase | arcC | -0.6 | 3.4E-02 |

| **Table S24.** Significantly (p ≤ 0.05) lower abundance proteins in *P. alloputida* KT2440 proteome in media containing glucose + 4-hydroxybenzoate with corresponding log_2_ fold change when compared with that in 4-hydroxybenzoate only. Only proteins with log_2_ FC ≥ 0.5 value are shown. | | | |
| --- | --- | --- | --- |
| **Protein name** | **Locus_tag** | **Log_2_ FC** | **p-value** |
| Dehydrogenase subunit, putative | PP_1661 | -3.3 | 2.5E-04 |
| Branched-chain amino acid ABC transporter, periplasmic amino acid-binding protein | PP_4867 | -3.1 | 2.4E-04 |
| Uncharacterized protein | PP_1659 | -2.8 | 9.4E-03 |
| 4-hydroxybenzoate transporter | pcaK | -2.6 | 2.4E-04 |
| Aldehyde dehydrogenase family protein | PP_5278 | -2.6 | 1.0E-02 |
| Electron transfer flavoprotein-ubiquinone oxidoreductase, putative | PP_4203 | -2.5 | 9.2E-03 |
| Branched-chain amino acid ABC transporter, periplasmic amino acid-binding protein | braC | -2.2 | 5.2E-06 |
| Xenobiotic reductase, putative | PP_1478 | -2.1 | 9.9E-05 |
| Hydrolase, isochorismatase family | PP_0711 | -2.0 | 9.4E-03 |
| OmpA family protein | PP_1121 | -1.9 | 4.7E-03 |
| Surface adhesion protein, putative | PP_0168 | -1.9 | 2.5E-04 |
| Acetolactate synthase, catabolic, putative | PP_1157 | -1.9 | 5.4E-03 |
| Uncharacterized protein | PP_1993* | -1.8 | 1.4E-02 |
| Elongation factor G 2 | fusB | -1.7 | 1.9E-04 |
| Uncharacterized protein | PP_0766 | -1.6 | 6.3E-03 |
| N-carbamoyl-beta-alanine amidohydrolase, putative | PP_4034 | -1.6 | 5.5E-03 |
| Uncharacterized protein | PP_3611 | -1.5 | 4.3E-03 |
| Uncharacterized protein | PP_2006 | -1.5 | 3.6E-03 |
| 4-hydroxybenzoate hydroxylase | pobA | -1.5 | 3.5E-03 |
| Cluster of Methyl-accepting chemotaxis transducer | PP_2310 | -1.5 | 1.1E-02 |
| General amino acid ABC transporter, periplasmic binding protein | aapJ* | -1.4 | 9.0E-03 |
| Isocitrate lyase | aceA | -1.4 | 1.5E-02 |
| Gamma-glutamyltransferase | ggt-2* | -1.4 | 3.1E-03 |
| Acetyl-coenzyme A synthetase 1 | acsA1 | -1.4 | 3.6E-03 |
| 3-carboxy-cis,cis-muconate cycloisomerase | pcaB | -1.3 | 1.7E-02 |
| ABC transporter, periplasmic binding protein | PP_1726 | -1.3 | 3.5E-03 |
| 3-oxoadipate CoA-transferase subunit B | pcaJ | -1.2 | 2.2E-02 |
| D-alanyl-D-alanine carboxypeptidase | dacA | -1.2 | 2.6E-03 |
| Protocatechuate 3,4-dioxygenase, beta subunit | pcaH | -1.2 | 1.6E-04 |
| DNA-binding protein HU-alpha | hupA* | -1.2 | 4.1E-03 |
| Polyhydroxyalkanoate granule-associated protein GA2 | PP_5007 | -1.1 | 4.8E-04 |
| Phosphate ABC transporter, periplasmic phosphate-binding protein, putative | PP_0824 | -1.1 | 2.5E-02 |
| Methylmalonate-semialdehyde dehydrogenase | mmsA-1 | -1.1 | 4.3E-03 |
| Glutamate synthase, small subunit, putative | PP_4037 | -1.0 | 2.3E-02 |
| Transcriptional regulator MvaT, P16 subunit, putative | PP_2947 | -0.9 | 2.6E-02 |
| Amino acid ABC transporter, periplasmic amino acid-binding protein | PP_0282 | -0.9 | 2.1E-03 |
| Uncharacterized protein | PP_2080 | -0.9 | 6.8E-03 |
| 5-aminovalerate aminotransferase DavT | davT | -0.8 | 1.7E-02 |
| Dipeptide ABC transporter, periplasmic peptide-binding protein | PP_0885* | -0.8 | 1.1E-02 |
| Transcriptional regulator MvaT, P16 subunit | PP_1366 | -0.8 | 4.7E-03 |
| Bifunctional purine biosynthesis protein PurH | purH | -0.8 | 1.1E-02 |
| Dihydroorotate dehydrogenase family protein | PP_4038 | -0.7 | 7.6E-03 |
| Uncharacterized protein | PP_3536 | -0.7 | 1.4E-02 |
| 2-isopropylmalate synthase | leuA | -0.7 | 1.9E-02 |
| Uncharacterized protein | PP_0397 | -0.6 | 7.1E-03 |
| Cysteine ABC transporter, periplasmic cysteine-binding protein, putative | PP_0227 | -0.6 | 3.1E-02 |
| Surface adhesion protein, putative | PP_0806 | -0.6 | 2.0E-02 |
| Catalase-peroxidase | katG | -0.6 | 6.4E-03 |
| Carbon starvation protein CstA | cstA* | -0.5 | 3.6E-02 |
| PhoH family protein | PP_1291 | -0.5 | 1.1E-02 |
| Protein HflC | hflC | -0.5 | 2.3E-02 |

| **Table S25.** Significantly (p ≤ 0.05) lower abundance proteins in *P. alloputida* KT2440 proteome in media containing glucose + ferulate with corresponding log_2_ fold change when compared with that in ferulate only. Only proteins with log_2_ FC ≥ 0.5 value are shown. | | | |
| --- | --- | --- | --- |
| **Protein name** | **Locus_tag** | **Log_2_ FC** | **p-value** |
| Quinoprotein ethanol dehydrogenase | qedH* | -4.8 | 3.1E-04 |
| Aldehyde dehydrogenase family protein | PP_2680 | -4.1 | 1.8E-04 |
| Isocitrate lyase | aceA | -3.4 | 2.5E-03 |
| Arylsulfatase, putative | PP_3352 | -3.3 | 3.6E-03 |
| Acyl-CoA dehydrogenase, ferrulic acid biotransformation protein, putative | PP_3354 | -3.1 | 4.6E-03 |
| Dehydrogenase subunit, putative | PP_1661 | -2.9 | 2.4E-05 |
| Branched-chain amino acid ABC transporter, periplasmic amino acid-binding protein | PP_4867 | -2.8 | 3.7E-04 |
| 4-hydroxybenzoate transporter | pcaK | -2.6 | 7.3E-05 |
| Vanillate demethylase A | vanA | -2.5 | 9.5E-05 |
| Acetyl-coenzyme A synthetase 1 | acsA1 | -2.3 | 9.7E-05 |
| N-carbamoyl-beta-alanine amidohydrolase, putative | PP_4034 | -2.2 | 1.2E-02 |
| Protocatechuate 3,4-dioxygenase, alpha subunit | pcaG | -2.1 | 3.4E-03 |
| Branched-chain amino acid ABC transporter, periplasmic amino acid-binding protein | braC | -2.0 | 3.8E-05 |
| 4-hydroxybenzoate hydroxylase | pobA* | -1.9 | 2.3E-04 |
| DNA-binding protein HU-alpha | hupA* | -1.9 | 2.0E-03 |
| Vanillin dehydrogenase | vdh | -1.9 | 4.1E-03 |
| Electron transfer flavoprotein-ubiquinone oxidoreductase, putative | PP_4203 | -1.8 | 1.8E-03 |
| Malate synthase G | glcB | -1.8 | 1.3E-03 |
| 3-carboxy-cis,cis-muconate cycloisomerase | pcaB* | -1.7 | 6.1E-05 |
| Vanillate O-demethylase oxidoreductase | vanB | -1.6 | 3.1E-04 |
| Beta-ketoadipyl CoA thiolase | pcaF | -1.6 | 7.7E-03 |
| Phosphate ABC transporter, periplasmic phosphate-binding protein, putative | PP_0824 | -1.6 | 2.6E-02 |
| Pyrroloquinoline-quinone synthase | pqqC* | -1.6 | 2.6E-04 |
| Feruloyl-CoA-synthetase | fcs | -1.5 | 7.6E-05 |
| General amino acid ABC transporter, periplasmic binding protein | aapJ* | -1.5 | 1.5E-03 |
| Acetolactate synthase, catabolic, putative | PP_1157 | -1.5 | 1.1E-02 |
| 3-oxoadipate CoA-transferase, subunit A | pcaI | -1.4 | 9.1E-03 |
| Enoyl-CoA hydratase/aldolase | ech | -1.4 | 1.1E-02 |
| Protocatechuate 3,4-dioxygenase, beta subunit | pcaH | -1.4 | 1.1E-02 |
| D-isomer specific 2-hydroxyacid dehydrogenase family protein | PP_1616 | -1.3 | 2.6E-03 |
| Uncharacterized protein | PP_3350* | -1.2 | 1.5E-03 |
| Glutamate synthase, small subunit, putative | PP_4037 | -1.2 | 1.1E-04 |
| Uncharacterized protein | PP_1659 | -1.1 | 4.8E-02 |
| Cluster of Cytochrome c oxidase, cbb3-type, subunit II | ccoO-1 | -1.1 | 1.6E-02 |
| RNA polymerase sigma factor RpoS | rpoS | -1.1 | 1.7E-02 |
| Elongation factor G 2 | fusB | -1.1 | 3.3E-03 |
| Uncharacterized protein | PP_0766 | -1.1 | 8.6E-05 |
| 3-oxoadipate enol-lactone hydrolase | pcaD | -1.0 | 6.9E-03 |
| Gamma-glutamyltransferase | ggt-2* | -1.0 | 7.4E-03 |
| Cluster of Methyl-accepting chemotaxis transducer | PP_2310 | -0.9 | 1.3E-02 |
| Uncharacterized protein | PP_2080 | -0.9 | 2.6E-03 |
| Oxygen-independent Coproporphyrinogen III oxidase family protein | PP_3781 | -0.9 | 1.1E-02 |
| Xenobiotic reductase, putative | PP_1478 | -0.9 | 3.3E-02 |
| Dipeptide ABC transporter, periplasmic peptide-binding protein | PP_0885* | -0.8 | 4.6E-02 |
| Cytochrome c-type protein | PP_2675* | -0.7 | 7.1E-03 |
| Transcriptional regulator MvaT, P16 subunit | PP_1366 | -0.5 | 3.1E-02 |

| **Table S26.** Significantly (p ≤ 0.05) lower abundance proteins in *P. alloputida* KT2440 proteome in media containing glucose + vanillate with corresponding log_2_ fold change when compared with that in vanillate only. Only proteins with log_2_ FC ≥ 0.5 value are shown. | | | |
| --- | --- | --- | --- |
| **Protein name** | **Locus_tag** | **Log_2_ FC** | **p-value** |
| Quinoprotein ethanol dehydrogenase | qedH* | -4.6 | 5.08E-04 |
| Aldehyde dehydrogenase family protein | PP_2680 | -3.6 | 2.80E-04 |
| Isocitrate lyase | aceA | -3.5 | 1.18E-04 |
| Branched-chain amino acid ABC transporter, periplasmic amino acid-binding protein | PP_4867 | -3.2 | 1.48E-04 |
| Acetyl-coenzyme A synthetase 1 | acsA1 | -2.9 | 5.22E-04 |
| 4-hydroxybenzoate hydroxylase | pobA* | -2.8 | 2.59E-04 |
| Vanillate demethylase A | vanA | -2.5 | 5.16E-05 |
| 3-carboxy-cis,cis-muconate cycloisomerase | pcaB* | -2.4 | 3.24E-03 |
| Beta-ketoadipyl CoA thiolase PcaF | pcaF | -2.3 | 1.36E-03 |
| Protocatechuate 3,4-dioxygenase, alpha subunit | pcaG | -2.0 | 8.05E-03 |
| Branched-chain amino acid ABC transporter, periplasmic amino acid-binding protein | braC | -1.7 | 1.21E-03 |
| Vanillate O-demethylase oxidoreductase | vanB | -1.6 | 1.60E-03 |
| Dehydrogenase subunit, putative | PP_1661 | -1.6 | 1.68E-02 |
| N-carbamoyl-beta-alanine amidohydrolase, putative | PP_4034 | -1.6 | 1.51E-04 |
| 4-hydroxybenzoate transporter | pcaK* | -1.5 | 1.19E-02 |
| Electron transfer flavoprotein-ubiquinone oxidoreductase, putative | PP_4203 | -1.5 | 3.70E-03 |
| Pyrroloquinoline-quinone synthase | pqqC* | -1.5 | 8.30E-03 |
| D-isomer specific 2-hydroxyacid dehydrogenase family protein | PP_1616 | -1.4 | 4.04E-03 |
| Protein RecA | recA | -1.4 | 3.16E-03 |
| Gamma-glutamyltransferase | ggt-2* | -1.4 | 3.60E-04 |
| DNA-binding protein HU-alpha | hupA* | -1.4 | 2.72E-02 |
| Phosphate ABC transporter, periplasmic phosphate-binding protein, putative | PP_0824 | -1.4 | 5.14E-03 |
| Malate synthase G | glcB | -1.3 | 1.26E-03 |
| Periplasmic binding protein, putative | PP_2676* | -1.3 | 3.77E-02 |
| Protocatechuate 3,4-dioxygenase, beta subunit | pcaH | -1.3 | 2.35E-02 |
| Ornithine decarboxylase | PP_0864 | -1.3 | 3.19E-02 |
| Uncharacterized protein | PP_1659 | -1.2 | 1.09E-03 |
| Cysteine desulfurase IscS | iscS* | -1.2 | 2.35E-02 |
| Oxidoreductase, aldo/keto reductase family | PP_3671* | -1.2 | 1.48E-03 |
| Transcriptional regulator MvaT, P16 subunit, putative | PP_3693 | -1.2 | 1.74E-02 |
| RNA polymerase sigma factor RpoS | rpoS | -1.2 | 1.74E-02 |
| 3-oxoadipate CoA-transferase subunit B | pcaJ* | -1.2 | 2.97E-02 |
| 3-oxoadipate CoA-transferase, subunit A | pcaI | -1.2 | 3.84E-03 |
| Quinoprotein ethanol dehydrogenase, putative | PP_2679* | -1.1 | 2.48E-03 |
| Uncharacterized protein | PP_0766 | -1.1 | 2.46E-03 |
| Peptidyl-prolyl cis-trans isomerase C | ppiC-2 | -1.1 | 8.35E-03 |
| Glutamate synthase, small subunit, putative | PP_4037 | -1.1 | 4.07E-04 |
| Uncharacterized protein | PP_2080 | -1.0 | 4.50E-04 |
| Transcriptional regulator MvaT, P16 subunit, putative | PP_2947 | -1.0 | 3.08E-02 |
| Cytochrome c-type protein | PP_2675* | -0.9 | 2.30E-02 |
| 2-isopropylmalate synthase | leuA | -0.9 | 1.99E-02 |
| Dihydroorotate dehydrogenase family protein | PP_4038 | -0.8 | 7.32E-03 |
| Transcriptional regulator MvaT, P16 subunit | PP_1366 | -0.8 | 6.81E-03 |
| Aromatic-amino-acid aminotransferase | tyrB-1 | -0.7 | 2.37E-02 |
| 3-oxoadipate enol-lactone hydrolase | pcaD | -0.7 | 3.41E-03 |
| Amino acid ABC transporter, periplasmic amino acid-binding protein | PP_0282 | -0.7 | 3.73E-02 |
| Major facilitator family transporter | PP_3740* | -0.6 | 2.58E-02 |
| Amino acid ABC transporter, periplasmic amino acid-binding protein | PP_1071 | -0.6 | 5.12E-03 |
| 30S ribosomal protein S6 | rpsF | -0.6 | 3.44E-02 |
| Alkyl hydroperoxide reductase AhpD | PP_2422* | -0.6 | 2.88E-02 |
| 50S ribosomal protein L24 | rplX | -0.5 | 4.35E-03 |

| **Table S27.** Differentially abundant proteins of crc-1 when compared with the control in media containing D-glucose + *p*-coumarate. Only proteins with log_2_ FC ≥ 0.5 and p ≤ 0.05 are shown. | | | |
| --- | --- | --- | --- |
| Protein name | Locus_tag | Log_2_ FC | p-value |
| **Higher Abundance Proteins** | | | |
| Ga0436255_01_1654516_1654920 | Ga0436255_01_1654516_1654920 | 1.3 | 8.5E-03 |
| amidophosphoribosyltransferase | Ga0436255_01_775291_776796 | 1.3 | 1.2E-02 |
| leucyl-tRNA synthetase | Ga0436255_01_1730904_1733510 | 1.2 | 1.7E-02 |
| NAD+ synthase | Ga0436255_01_3041191_3042018 | 0.9 | 3.0E-02 |
| nucleotide-binding universal stress UspA family protein | Ga0436255_01_4357332_4358195 | 0.9 | 1.1E-02 |
| 2-Keto-3-deoxy-6-phosphogluconate aldolase | Ga0436255_01_3544702_3545376 | 0.8 | 3.7E-02 |
| 3-isopropylmalate/(R)-2-methylmalate dehydratase large subunit | Ga0436255_01_789961_791394 | 0.8 | 2.0E-02 |
| pyruvate carboxylase subunit B | Ga0436255_01_2523387_2525195 | 0.8 | 1.0E-02 |
| glutamyl-tRNA synthetase | Ga0436255_01_800516_801997 | 0.8 | 2.2E-02 |
| branched-chain amino acid transport system substrate-binding protein | Ga0436255_01_1169501_1170616 | 0.7 | 1.7E-02 |
| peptidyl-prolyl cis-trans isomerase SurA | Ga0436255_01_2896045_2897364 | 0.7 | 2.0E-02 |
| aldehyde dehydrogenase | Ga0436255_01_1761115_1762635 | 0.7 | 2.9E-02 |
| glutamate dehydrogenase (NADP+) | Ga0436255_01_3214533_3215873 | 0.7 | 4.0E-02 |
| ATP-dependent Clp protease ATP-binding subunit ClpX | Ga0436255_01_569630_570913 | 0.7 | 3.5E-02 |
| 3-isopropylmalate/(R)-2-methylmalate dehydratase small subunit | Ga0436255_01_789320_789964 | 0.6 | 1.3E-02 |
| glutamine synthetase | Ga0436255_01_1966094_1967500 | 0.5 | 5.7E-03 |
| **Lower Abundance Proteins** | | | |
| hypothetical protein | Ga0436255_01_2323954_2324595 | -1.5 | 3.1E-02 |
| peroxiredoxin (alkyl hydroperoxide reductase subunit C | Ga0436255_01_1214403_1215005 | -1.0 | 3.7E-03 |
| elongation factor Tu | Ga0436255_01_2940070_2941263 | -0.6 | 1.6E-02 |
| molecular chaperone IbpA | Ga0436255_01_795880_796323 | -0.6 | 2.2E-02 |

## **FIGURES**

**Figure S1.** Phenotype of *P. putida* M2 showing OD_600_ and substrate consumption in a minimal medium containing sugar only (a, b) and aromatic compound only (c, d). The data represent mean value of three replicates and error bars represent standard deviation.

**Figure S2.** Phenotype of *P. putida* M2 (a-b) and *P. alloputida* KT2440 (c) showing OD_600_ in logarithmic scale in a mixture of sugar and aromatic compound as carbon sources. The data represent the mean value of three replicates and error bars represent the standard deviation.

**Figure S3.** Phenotype of *P. putida* M2 showing OD_600_ (a, d) and substrate consumption in a mixture of glucose + 4-hydroxybenzoate (b) glucose + vanillate (c) xylose + 4-hydroxybenzoate (e) xylose + vanillate (f) as carbon sources. The data represent mean value of three replicates and error bars represent standard deviation.

**Figure S4.** The significantly (p ≤ 0.05, log_2_FC ≤ 0.5) lower abundance upper pathway proteins (a) and β-ketoadipate pathways proteins (b) involved in aromatic catabolism in *P. putida* M2 grown in dual carbon sources conditions when compared to the reference condition (aromatics only).

**Figure S5.** The significantly (p ≤ 0.05, log_2_FC ≤ 0.5) lower abundance upper pathway proteins (a) and β-ketoadipate pathways proteins (b) involved in aromatic catabolism in *P. alloputida* KT2440 grown in dual carbon sources conditions when compared to the reference condition (aromatics only).

**Figure S6.** Growth kinetic analysis of *P. putida* M2 carrying pRGPspdCas9bad-edd (blue) showing delayed growth to verify the sgRNA expression and thus demonstrating the functionality of the CRISPRi system. The growth was compared to control *P. putida* M2 carrying pRGPspdCas9bad with no targeting sgRNA sequence (red). According to Gauttam et al (1) paper, pRGPspdCas9bad-edd1 was designed to down-regulate the expression of *edd* gene, an essential gene for growth on glucose, in *P. putida.* The growth was assessed in minimal media containing glucose (1% w/v) as carbon source in the presence of inducers IPTG (1 mM) and varying arabinose concentration of 0.1% w/v (a) 0.2% w/v, and (b) 0.5% w/v (c). Comparable growth was observed at all arabinose concentrations. The data represents mean value of three replicates and error bars represent standard deviation.

**Figure S7.** Growth performance (Phenotype) of *P. putida* M2 CRISPRi and wild-type strains showing OD_600_ and substrate consumption in a mixture of glucose + *p*-coumarate (a, b, c), glucose + Ferulate (d, e, f), and xylose + *p*-coumarate (g, h, i) as carbon source. The data represent the mean value of three replicates and error bars represent the standard deviation.

**Figure S8.** Growth performance (Phenotype) of *P. putida* M2 CRISPRi and wild-type strains showing OD_600_ in logarithmic scale in a mixture of glucose + *p*-coumarate (a), glucose + Ferulate (b), and xylose + *p*-coumarate (c) as carbon source. The data represent the mean value of three replicates and error bars represent the standard deviation.

**Figure S9.** Concentration profile of arabinose, the inducer used for sgRNA expression, during growth curve experiment comparing the phenotype of CRISPRi strains with the wild-type when grown in a mixture of glucose + *p*-coumarate (a) glucose + ferulate (b) and xylose + *p*-coumarate (c).

**Figure S10.** Design of sgRNA coding sequences. The position of sgRNA target in the crc gene (a). Three different 20 base pair sgRNAs; crc-1 (b) crc-2, and (c) crc-3 (d) were designed to target the crc gene using dual-inducible *Streptococcus pyogenes* spdCas9-based CRISPRi system. The sgRNA scaffold sequences for dCas9 binding are underlined.

## **References**

1. Gauttam R, Mukhopadhyay A, Simmons BA, Singer SW. 2021. Development of dual-inducible duet-expression vectors for tunable gene expression control and CRISPR interference-based gene repression in *Pseudomonas putida* KT2440. Microb Biotechnol 14:2659–2678.
